# Cost efficiency versus energy utilization in green ammonia production from intermittent renewable energy

**DOI:** 10.1038/s44286-025-00207-9

**Published:** 2025-04-18

**Authors:** Collin Smith, Laura Torrente-Murciano

**Affiliations:** Department of Chemical Engineering and Biotechnology, University of Cambridge, Cambridge, UK

**Keywords:** Chemical engineering, Renewable energy

## Abstract

Electrification of the chemical industry with renewable energy is critical for achieving net zero goals and the long-term storage of renewable energy in chemical bonds, particularly carbon-free molecules such as ammonia. Through an analysis of green ammonia production with solar and wind energy at more than 4,500 locations across Europe, this work demonstrates that maximizing cost efficiency is decoupled from maximizing energy utilization due to the intermittency of renewable energy. By devising the metric of levelized cost of utilization, the economic drive for energy curtailment is connected to the high cost to utilize portions of solar or wind energy profiles with unequal seasonal distribution. Combining solar and wind energy or ramping production decreases the cost of utilizing energy, thereby decreasing curtailment. A framework for evaluating the power-to-x economics within the context of electricity grids is illustrated using the value of utilizing energy, which indicates that electrified chemicals production is an attractive market for renewable energy at locations with high penetration on the grid.

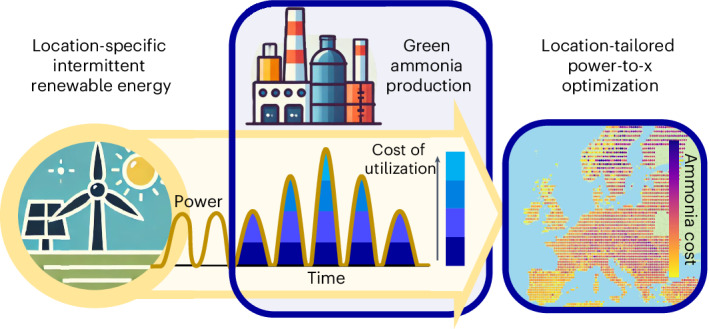

## Abstract (web summary)

The synthesis of chemicals through power-to-x economics is critical to achieve net zero goals, but is limited by the challenge of designing processes powered by intermittent renewable energy. Here the true cost of utilizing intermittent energy is assessed and the economic drivers for energy curtailment are revealed.

## Main

Ammonia production through the Haber–Bosch (HB) process has been at the center of the chemical sector since it was industrialized at the beginning of the twentieth century, becoming one of the most produced chemicals^1^ and epitomizing the uniting of the chemicals industry with fossil fuels. Approximately 2% of global CO_2_ emissions and ~45% of emissions in the primary chemical sector are attributed to ammonia production (that is, brown ammonia)^2^, which provides nitrogen fertilizer to feed a growing global population. In recent years, green ammonia production using renewable solar or wind energy has garnered accelerated attention because the high energy density of ammonia in liquid form (12.7 MJ l^−1^) offers the potential to address the issues of renewable energy storage, transport and use as a fuel^1,3–7^.

Although technologically feasible, green ammonia production has remained an economic challenge due to the existing integration of the HB process loop with fossil-fuel reforming for hydrogen production^8^. In the electrified HB process, hydrogen and nitrogen are generated through water electrolysis and air separation, respectively, and therefore, the process needs to be holistically reoptimized^9^. Of principal importance is the shift from the continuous supply of coal/natural gas in brown ammonia production to intermittent solar or wind energy, which is misaligned with the HB process loop originally designed to operate continuously. This has led to novel methods for flexible ammonia production^10–13^, or process designs with intermediate hydrogen/electricity buffer storage or backup power from grid electricity^14–16^.

The techno-economics of producing green ammonia is an extensive research field, which has been described in several reviews^17–19^. Across the literature, it is generally assumed that the primary result of an optimization model is the prescribed cost for producing green ammonia, particularly compared with historical ammonia prices, and often includes projections for future cost reductions^20,21^. The production costs of green ammonia are highly geographically dependent^16,20,22^, with case studies normally focused on locations with plentiful renewable energy (that is, high capacities)^14,15,23–27^. These studies have indicated that combining solar and wind energy or allowing for a flexible HB process can decrease the cost of ammonia through better alignment between the supply of renewable energy and the consumption of energy in the HB process. Additionally, connection to the grid is often included in models of so-called on-grid ammonia production to either provide backup power or consistent energy supply as optimal^15,25,28–32^, but it can also result in increased carbon intensity^33^. Connecting high-load chemical processes to the grid naturally increases the grid’s base-load demand, which increases the required capacity of dispatchable fossil power. As a consequence, powering chemical process with the grid could undermine decarbonization targets by perpetuating the need for fossil fuels; this study, therefore, focuses on truly green off-grid ammonia production.

The intentional underutilization of energy supply, known as curtailment, has become a prevalent topic in the analyses of electrical grids, particularly with increasing renewable energy penetration^34–37^. Similarly, the benefit of curtailment to the economics of ammonia production has been demonstrated in some studies, and its extent has been noted in connection to electricity price^16,20^, electrolyzer cost^38^, hydrogen storage cost^24^, connection to the grid^32^ and HB process flexibility^23^. However, curtailment is typically discussed as an ancillary result, and in other cases, is either not presented or not included in the model^21,27,39–41^. In some models, energy is only curtailed when energy supply exceeds the capacity of the process to intake power^15,26,42^, thereby misrepresenting the economic drivers for curtailment. Likewise, the lack of analysis dedicated to curtailment across a wide range of scenarios and locations has led to curtailment being implicitly assumed without its importance and magnitude being fully quantified.

In power systems, the curtailment of solar/wind energy is often associated with the value of renewable electricity, which is strongly dependent on when the energy is produced because the selling price for electricity on the grid changes with time based on the merit-order curve and the supply of energy from renewables with zero operating costs^43^. The importance of temporal dependence for the value of renewables connected to the grid has led to metrics such as levelized avoided cost of energy^44^, cost of valued energy^45^, system levelized cost of energy^46,47^ and market value^48^. However, similar analyses and metrics have not been applied to the temporal cost or value of renewable electricity powering a chemical plant and, therefore, electrification of the chemicals sector has been lacking continuity with the existing economic assessments concerning electrical grids.

In this work, through the optimization of green ammonia production, we demonstrate that cost minimization (that is, cost efficiency) is decoupled from the maximization of energy utilization (that is, curtailment) when using intermittent renewable energy rather than fossil fuels for chemical process design. The widespread economic advantage of intentionally underutilizing energy is shown with off-grid solar and wind energy across the European geography (between 33.5° N and 65° N and 10° W and 40° E). By defining the levelized cost of utilization (LCOU) as a metric for analyzing differential portions of the renewable energy supply profiles, we unveil the process components driving energy curtailment as a function of location. Integrating solar and wind energy or ramping the HB process reduces the cost of utilizing energy and, consequently, reduces the cost of ammonia, but rarely removes the need for curtailment. Additionally, we quantify the levelized value of utilization (LVOU) for ammonia production within the context of existing electrical grids. This framework demonstrates that electrified chemicals production can be a profitable way of utilizing renewable energy in locations with low market value of renewables caused by their high penetration in the grid.

## Results

### Cost efficiency versus energy utilization

**Extended Data Fig. 1:**
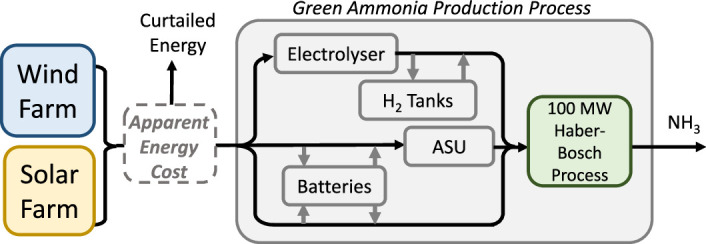
Schematic of off-grid green ammonia production system. The temporal energy variation of solar and wind energy for a given location is included on an hourly basis for each profile. The apparent cost to utilize energy determines the amount of energy curtailed to reach the economically optimal utilization of energy.

The optimized capacity planning of a green ammonia production process using solar and wind energy (Extended Data Fig. 1) shown in this section challenges the conventional emphasis that increased energy utilization (that is, energy efficiency) corresponds to a decrease in cost. Energy intermittency, particularly on a seasonal timescale, causes a departure from traditional optimization due to the need for an intermediate buffer in the form of hydrogen/battery storage to align intermittent energy supply with constant ammonia production.

**Fig. 1:**
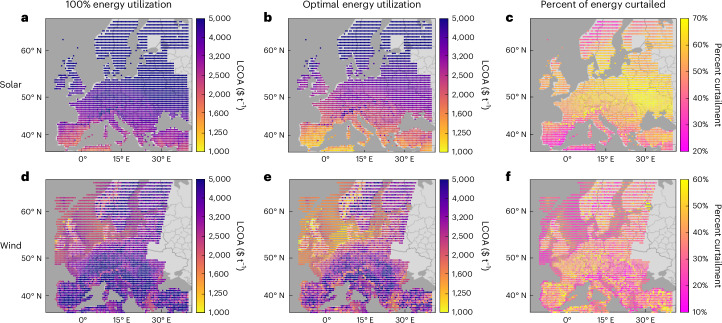
Optimized cost and energy utilization of constant ammonia production (100 MW). **a**, Solar energy assuming 100% energy utilization (that is, no curtailment). **b**, Solar energy with optimized energy utilization. **c**, Optimal amount of curtailment with solar energy. **d**, Wind energy assuming 100% energy utilization. **e**, Wind energy with optimized energy utilization. **f**, Optimal amount of curtailment with wind energy. Source data

Utilizing 100% of the available solar/wind energy in the HB process continuously producing ammonia results in prohibitively high levelized cost of ammonia (LCOA), defined as the annualized capital and operating costs normalized by the total ammonia production, as shown in Fig. 1a,d for solar and wind energy, respectively. Once energy supply is utilized to an optimal degree (that is, curtailed at an optimal level) for each location, there is a marked decrease in the LCOA (Fig. 1b,e). The best 2% of locations exhibit an LCOA below $1,240 t^−1^ and $1,250 t^−1^ for solar and wind, respectively. This cost is well above the typical market price of fossil-fuel-derived ammonia ($400–700 t^−1^)^49^ and estimates previously reported in the literature, because this analysis considers the most conservative assumption of the HB process with constant production.

Although the general distribution of the best solar locations in the Mediterranean South and the best wind locations around the North Sea is as anticipated, the optimal amount of curtailment is exceptional, as shown in Fig. 1c,f for solar and wind, respectively. The results clearly demonstrate that to sustain constant ammonia production, it is economically optimal to curtail a sizable amount of energy, averaging 53% and 34% for solar and wind energy, with ranges of 30–68% and 18–55%, respectively. Solar energy tends to be curtailed more than wind because solar panels are cheaper than wind turbines. It should be noted that the energy curtailed with respect to ammonia production does not need be wasted, but rather could be utilized elsewhere, making sector coupling particularly relevant. Nevertheless, for ammonia production alone, it is markedly optimal to intentionally underutilize energy, demonstrating a shift in process design, which has historically emphasized maximizing the energy efficiency to decrease cost when using a continuous supply of (fossil) energy.

**Extended Data Fig. 2:**
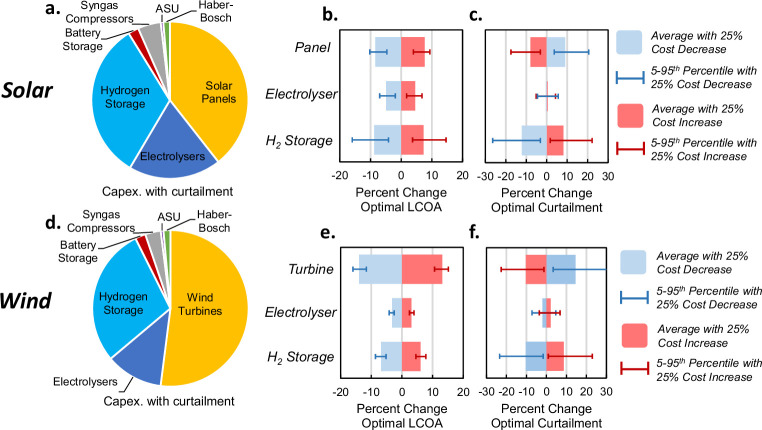
Sensitivity analysis of LCOA and optimal percent curtailment for constant ammonia production (100MW). **a**. Contribution of process components to the capital cost with optimal percent curtailment of solar energy averaged across all locations. **b**. Percent change in LCOA with solar energy. **c**. Percent change in optimal curtailment with solar energy. **d**. Contribution of process components to the capital cost with optimal percent curtailment of wind energy averaged across all locations. **e**. Percent change in LCOA with wind energy. **f**. Percent change in optimal curtailment with wind energy. Results represent the average over all locations, with the absolute range of the 5–95^th^ percentiles in LCOA and curtailment across locations represented by error bars. Source data

A sensitivity analysis with respect to the cost of major process components (Extended Data Fig. 2) indicates that the LCOA is highly sensitive to the cost of solar panels/wind turbines and hydrogen storage. By contrast, the sensitivity to electrolyzer cost is less than expected based on literature reports, because a constant HB process is dominated by hydrogen storage to balance long-term energy fluctuations. Of particular note is the sensitivity of optimal curtailment to the cost of major components. Increasing panel/turbine cost presents a marked decrease in the optimal amount of curtailment, and vice versa, as indicated previously^20,38^. Similarly, there is a marked sensitivity of optimal curtailment to hydrogen storage cost such that higher cost leads to more curtailment. In the case of electrolyzer cost, it has a variable effect on optimal curtailment based on the location because a cheaper electrolyzer facilitates the utilization of ‘peaks’ in energy supply during periods of overall energy deficit, thereby promoting increased turbine/panel capacity and, therefore, more overall curtailment during periods of energy surplus.

### LCOU

**Extended Data Fig. 3:**
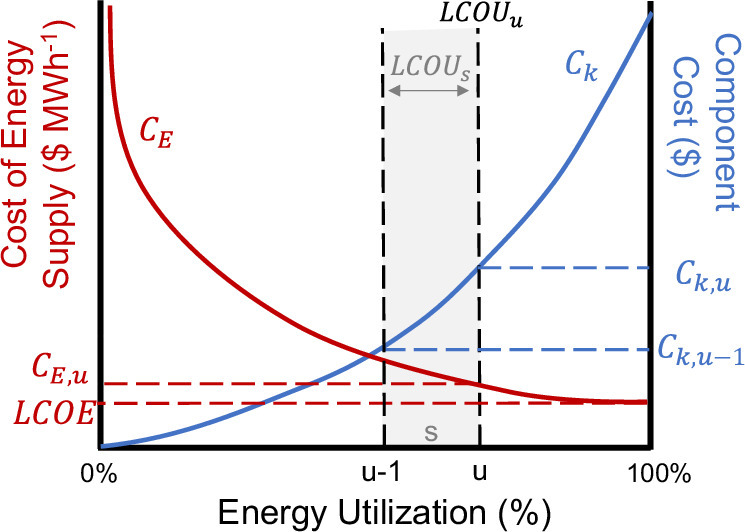
Schematic of energy and process costs as a function of percent energy utilization. The cost (*C*_*k*_) of process component *k* increases with percent energy utilization. The cost of energy supply per energy utilized (*C*_*E*_) in $ MWh^−1^ decreases as energy utilization increases, until at 100% utilization it reaches the LCOE of solar panels or wind turbines. The LCOU_u_ is the cost of utilizing energy up to percent utilization (*u*) which graphically corresponds to the area under the LCOU_s_ curve divided by the percent utilization. The LCOU_s_ is the cost of utilizing the energy in stratum (*s*), which is the energy between percent utilization (*u*) and (*u*-1).

To investigate the economic drivers for curtailment, the LCOU ($ MWh^−1^) for an aggregate of energy is defined as the change in the cost of process components required to utilize energy normalized by the amount of energy (equation (14)). This metric is applied to (1) strata of energy (*s*) consisting of 1% increments in energy in the total solar or wind energy profile (LCOU_*s*_) and (2) all energy up to a percent utilization (*u*; LCOU_*u*_), as represented in Extended Data Fig. 3. As a result, LCOU_*s*_ represents the differential cost of utilizing energy at a given percent utilization. Additionally, the levelized apparent energy cost (LAEC_*u*_) is defined as the cost of utilization (LCOU_*u*_) plus the cost of energy supply with solar panels or wind turbines. These metrics are assessed at a range of locations by randomly selecting ten of the best, average and worst locations for solar or wind energy. The best, average and worst locations are defined as being in the lowest 10%, 45th–55th percentile and the highest 10% of the LCOA, respectively (Supplementary Table 5 provides the list of locations selected). The results are averaged across the locations and years in each category to demonstrate general trends and highlight the efficacy of evaluating the LCOU for elucidating the economics of green ammonia production.

**Fig. 2:**
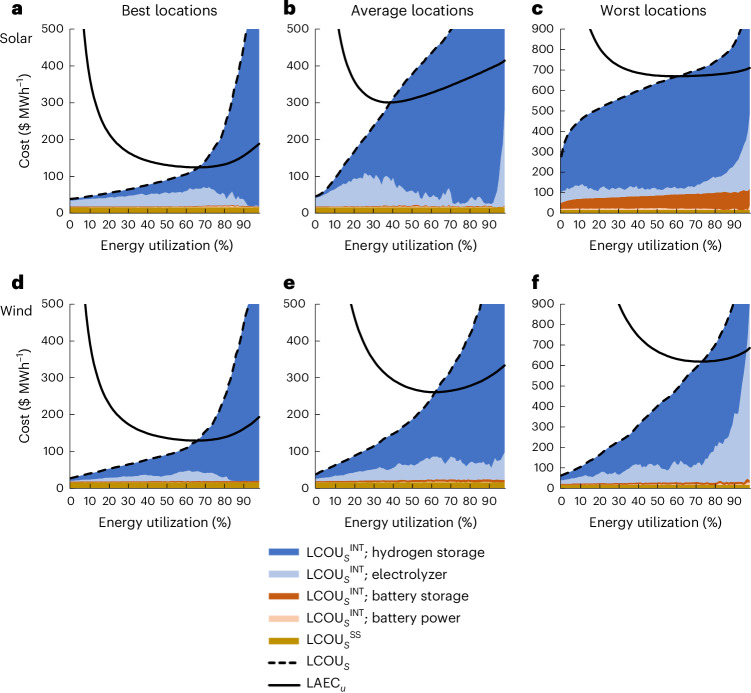
Energy utilization cost and total cost as a function of percent utilization for solar/wind profiles. **a**–**f**, Sampled locations are divided into the best solar (**a**) and wind (**d**) locations (lowest 10% of the LCOA), the average solar (**b**) and wind (**e**) locations (45th–55th percentile of the LCOA), and the worst solar (**c**) and wind (**f**) locations (highest 10% of the LCOA). The cost of utilizing a stratum of energy (LCOU_*s*_) is divided into the steady-state process components (LCOU_*s*_^SS^) and the components that accommodate energy intermittency (LCOU_*s*_^INT^). LAEC_*u*_ is the total cost at a percent utilization, which includes the cost of energy supply and LCOU_*u*_. The results are averaged across the locations and years in each category of solar or wind energy. Battery power refers to the capacity to exchange electricity (MW), whereas battery storage refers to the electrical capacity (kWh). Source data

The cost to utilize a stratum of energy (LCOU_*s*_) and total apparent cost (LAEC_*u*_) as a function of percent energy utilization is shown in Fig. 2 for profiles of solar or wind power in each locational category. The LCOU_*s*_ value is divided into the cost of components needed to convert electricity to ammonia at the steady state (LCOU_*s*_^SS^) if the energy is available consistently, and the added cost of process components required due to the intermittency of the energy stratum (LCOU_*s*_^INT^), as shown in Fig. 2 (colored areas). For clarity, one should note that LCOU_*u*_ is the average LCOU_*s*_ value up to *u* (Methods and equation (16)), which graphically corresponds to the area under the LCOU_*s*_ curve divided by the percent utilization. When including the cost of energy supply, LAEC_*u*_ (Fig. 2, solid line) approaches infinity at zero energy utilization and decreases with increasing energy utilization until it crosses the LCOU_*s*_ value. At the intersection of LAEC_*u*_ and LCOU_*s*_, the added cost of utilizing additional strata of energy is greater than the total cost of the energy up to the intersection point. Therefore, this intersection point corresponds to the optimal level of energy utilization (and curtailment) and the minimum LAEC_*u*_ value. For reference, a cost of $1,000 t^−1^_NH3_ translates to ~$95 MWh^−1^, and, therefore, the intersection of LAEC_*u*_ and LVOU_*s*_ at >$95 MWh^−1^ aligns with the best locations having an LCOA of >$1,000 t^−1^_NH3_.

When comparing solar and wind energy (Fig. 2), it is clear that the LCOU_*s*_ is strongly dependent on the location as well as percent energy utilization. Across all the locations, the cost to utilize energy at the steady state (LCOU_*s*_^SS^) is constant for all strata of energy and is small (~$16 MWh^−1^) compared with the utilization cost stemming from energy intermittency (LCOU_*s*_^INT^). For the best solar locations (Fig. 2a), most of the energy has relatively small LCOU_*s*_^INT^, which generates a utilization cost of $75 MWh^−1^ for the optimally utilized energy (LCOU_*u*__=opt_). The LCOU_*s*_^INT^ value at low-energy strata is dominated by the added electrolyzer cost, which must be oversized to make use of energy in pronounced daily cycles. Only at higher strata does the LCOU_*s*_^INT^ value become dominated by the hydrogen storage cost because the energy in the stratum has unequal distribution over a year, becoming more concentrated during the summer months. As a result, the excessive cost to utilize high-energy strata leads to curtailing this portion of the energy profile. A similar trend of utilization cost with the stratum is apparent for the best wind locations (Fig. 2d), with the exception that hydrogen storage contributes more to the LCOU_*u*__=opt_ value because wind is less consistently available on a daily basis and, therefore, storage is required to utilize even low-energy strata.

For locations with average solar or wind energy (Fig. 2b,e), the LCOU_*s*_ value increases more rapidly with percent utilization. The average energy profiles have more short- and long-term energy fluctuations, leading to greater electrolyzer and hydrogen storage cost, respectively. Consequently, the optimal utilization cost LCOU_*u*__=opt_^INT^ is much higher for both solar ($158 MWh^−1^) and wind ($112 MWh^−1^) locations, indicating that ammonia will cost more than $1,000 t^−1^_NH3_ solely as a result of the intermittency of solar or wind power. In both cases, LCOU_*s*_ intersects LAEC_*u*_ at a lower optimal percent utilization (that is, higher optimal curtailment).

In the worst solar locations (Fig. 2c), even the lowest stratum (1% energy utilization) requires hydrogen storage to utilize the energy because the energy profile includes long periods with little energy produced. For higher-energy strata, the change in LCOU_*s*_ is shallower compared with other locations because the energy profile approximates a square pulse function in the summer months. This trend, as well as the higher cost of energy supply included in the LAEC_*u*_, leads to higher optimal percent utilization compared with the average locations. Unlike the worst solar locations, the worst wind locations (Fig. 2f) have a similar shape to average wind locations (note the different axis scales), indicating a similar energy profile shape.

The analysis of energy cost demonstrated in Fig. 2 illustrates the disproportionate impact of hydrogen storage on the cost of utilizing energy compared with electrolyzers, especially battery power/storage. As a result, it highlights the economic drivers that determine the cost of utilizing energy in different strata of a renewable energy profile and, therefore, provides insights into the factors influencing curtailment as a function of location. Further, the analysis of apparent energy cost (Fig. 2) underscores that the minimization of cost is not analogous with the maximization of energy utilization because intermittent energy supply generates additional costs for utilizing different portions of the energy supply. When powering a chemical process with fossil fuels (for example, methane), the cost of utilizing energy is described by LCOU_*s*_^SS^, which has no dependence on percent utilization, and therefore, LAEC_*u*_ reaches a minimum at 100% utilization. However, in the case of an electrified chemicals process, the cost of utilizing energy is dominated by the influence of energy intermittency (LCOU_*s*_^INT^ ≫ LCOU_*s*_^SS^), and therefore, LAEC_*u*_ is minimized by curtailing expensively intermittent energy.

### Combining solar and wind energy

**Fig. 3:**
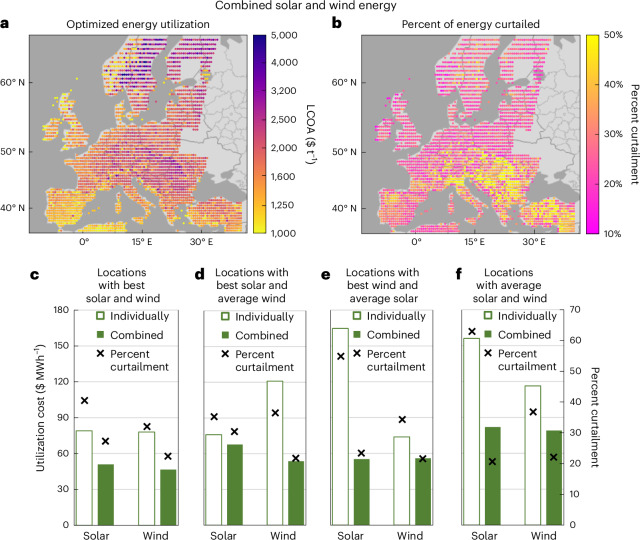
Effect of integrating solar and wind energy for constant ammonia production (100 MW). **a**, LCOA with optimized capacity of solar panels, wind turbines and curtailment at each location. **b**, Optimal percent curtailment. **c**–**f**, Average cost of utilizing energy at the optimal percent utilization (LCOU_*u*__=opt_) and average optimal percent curtailment for solar and wind energy either individually or combined for ten sampled locations in different locational categories. **c**, Best solar and wind (lowest 15% of the LCOA for each individually). **d**, Best solar (lowest 10% of the LCOA) and average wind (45th–55th percentile of the LCOA). **e**, Best wind (lowest 10% of the LCOA) and average solar (45th–55th percentile of the LCOA). **f**, Average solar and wind (45th–55th percentile of the LCOA for each). Locations are listed in Supplementary Table 6. Results are averaged across the individual locations and years. Source data

**Extended Data Fig. 4:**
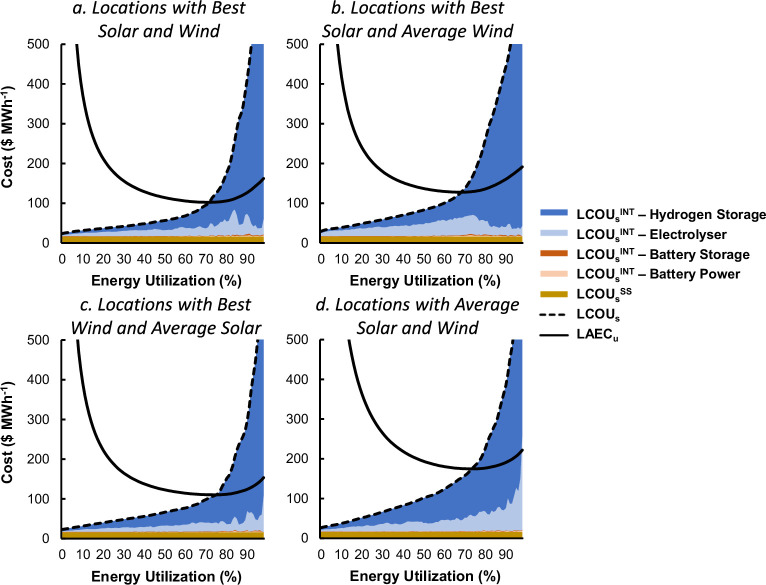
Energy utilization cost as a function of percent utilization when combining solar and wind. The categories are (**a**) locations with the best solar and wind (lowest 15% of LCOA for each), (**b**) locations with the best solar (lowest 10% of LCOA) and average wind (45^th^–55^th^ percentile of LCOA), (**c**) locations with the best wind and average solar and (**d**) locations with average solar and wind (45^th^–55^th^ percentile of LCOA for each). The results for 10 locations sampled in each category are averaged. It is assumed that the ratio of solar to wind remains constant at each level of energy utilization, and this ratio is determined by the optimal ratio at the optimal level of curtailment. Source data

Having demonstrated that curtailment decreases the cost of producing ammonia from renewable solar or wind energy, this section assesses the decrease in LCOA and optimal curtailment when combining solar and wind energy and analyzes the change in LCOU at the sample locations. As shown in Fig. 3a, by combining solar and wind, there is a widespread decrease in LCOA across Europe (average LCOA, $1,820 t^−1^), compared with solar ($3,330 t^−1^) and wind ($2,820 t^−1^) individually, with the best 2% of locations now below $1,030 t^−1^. The geographic distribution of solar/wind prevalence follows a South/North trend (Supplementary Fig. 2a) and the percent curtailment decreases across Europe (Fig. 3b), with the greatest decrease of ~30–60% occurring when there is approximately equal balance of solar and wind energy (Supplementary Fig. 2c). Additionally, it is important to note that the average curtailment only decreases to 29% (within a range of 17–51%) when integrating solar and wind energy because the variations in solar and wind energy are not perfectly out of phase and the LCOU_*s*_ value remains pronounced for the highest-energy strata (Extended Data Fig. 4).

The economic benefit of combining solar and wind energy is further demonstrated in Fig. 3c–f through the LCOU_*u*__=opt_ for solar or wind energy at different locational categories when solar and wind energy are combined compared with the individual energy supplies. For locations with best solar and wind (lowest 15% of the LCOA for each), combining the energy profiles notably decreases the cost of utilizing solar or wind energy in equal portions (Fig. 3c) due to the temporal complementarity in the energy profiles. Conversely, at locations in which either the solar or wind energy is average quality (45th–55th percentile of the LCOA) and the corresponding energy supply at the location is the best for solar or wind (lowest 10% of the LCOA), the LCOU_*u*__=opt_ value of the average profile is particularly improved. The LCOU_*u*__=opt_ value decreases from $120 MWh^−1^ to $55 MWh^−1^ for average wind energy (Fig. 3d), and from $165 MWh^−1^ to $55 MWh^−1^ for average solar energy (Fig. 3e). Indeed, the LCOU_*u*__=opt_ value for the average solar/wind supply at these locations is lower than the corresponding best solar/wind supply at the location. This reversal in LCOU is a consequence of the average energy supply reducing the cost of utilizing energy in the best energy supply by displacing the need for hydrogen storage and maximizing the average operating capacity of the electrolyzer. In other words, the average energy supply is providing mainly ‘baseline’ power and is, therefore, disproportionally represented in low-energy strata, which have lower LCOU_*s*_^INT^ values (Extended Data Fig. 4). As a result, the optimal percent curtailment decreases more for the average solar/wind supply compared with the best solar/wind supply at these locations (Fig. 3d,e).

Similarly, at locations with both average solar and wind energy (Fig. 3f), combining the energy profiles generates particularly lower LCOU_*u*__=opt_ values for both. Although the absolute change in cost is less than the average solar/wind locations (Fig. 3d,e), energy is sourced in approximately equal proportion from solar and wind (Fig. 3f; averaging 40% solar energy), as opposed to mostly solar or wind (Fig. 3d,e; 82% solar and 78% wind, respectively). As a result, the total cost reduction is greater at locations with average supplies of both solar and wind energy because the temporal complementarity of the energy profiles reduces the LCOU_*s*_ values of both energy profiles, generating a particularly different trend of LCOU_*s*_ as a function of percent utilization (Extended Data Fig. 4). Therefore, it can be noted that the cost of utilizing average supplies of solar/wind energy markedly decreases when it is supplementing another energy supply.

### Ramping the HB process

The temporal adjustment of ammonia production levels through ramping the HB process is widely considered to be beneficial to the economics of green ammonia production^14^. In this section, the effect of ramping on decreasing the LCOA and curtailment is analyzed at locations across Europe and the impact of ramping on the LCOU is evaluated for a subset of locations. A 60% minimum operating capacity is the baseline ramping range, with the benefit of further flexibility analyzed for a subset of locations.

**Fig. 4:**
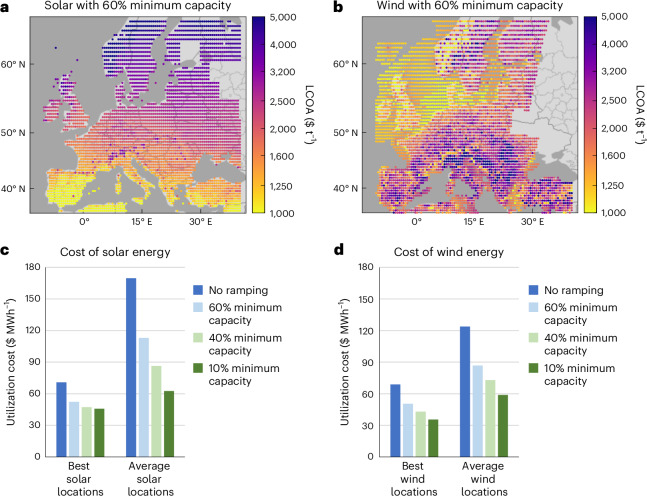
Effect of ramping the HB process for ammonia production with solar or wind energy. **a**,**b**, LCOA with solar (**a**) and wind (**b**) energy and optimal ramping of the HB process between 60% and 100% capacity. **c**,**d**, Average cost of utilizing energy at the optimal percent utilization (LCOU_*u*__=opt_) for samples of ten locations of solar (**c**) or wind (**d**) energy as a function of the minimum operating capacity and category of energy supply at a location. The best solar/wind locations are in the lowest 10% of the LCOA without ramping and the average solar/wind locations are in the 45th–55th percentile of the LCOA without ramping. Source data

The effect of ramping production between 60% and 100% of the installed HB capacity on the LCOA using solar or wind energy with the optimal amount of curtailment is shown in Fig. 4a,b. The best 2% of locations present an LCOA value of $950 t^−1^ and $925 t^−1^, respectively, which is a notable reduction compared with that without HB ramping. However, the average LCOA with ramping ($2,380 t^−1^ and $2,180 t^−1^, respectively) is greater than that with combined solar and wind ($1,820 t^−1^), indicating that combining solar and wind is more universally beneficial than ramping in this case. Ramping the HB process also decreases the average curtailment to 39% (range, 11–57%) and 20% (range, 4–41%) for solar and wind, respectively (Supplementary Fig. 3).

To further highlight the economic impact of ramping the HB process, the average cost of utilizing energy at the optimal percent utilization (LCOU_*u*__=opt_) is presented in Fig. 4c,d for solar or wind, respectively, as a function of decreasing the minimum capacity of the HB process for samples of ten locations. As the ammonia production level is ramped to follow the energy profile, the LCOU_*u*__=opt_ value decreases depending on the location. At the best solar or wind locations, the cost of utilizing energy decreases by ~$10–$20 MWh^−1^ with decreased minimum HB capacity. However, the LCOU_*u*__=opt_ value of the best solar energy plateaus, indicating diminishing returns for increased process flexibility. Similarly, the cost for utilizing energy in average solar or wind locations decreases by up to $50 MWh^−1^ when decreasing the minimum HB capacity, thereby narrowing the disparity between the best and average locations. Still, with 10% minimum operating capacity, the LCOU_*u*__=opt_ value at locations of average solar or wind energy is ~$60 MWh^−1^ (of which ~$44 MWh^−1^ is LCOU_*u*__=opt_^INT^), demonstrating a notable cost for utilizing the energy even with a highly flexible HB process.

**Fig. 5:**
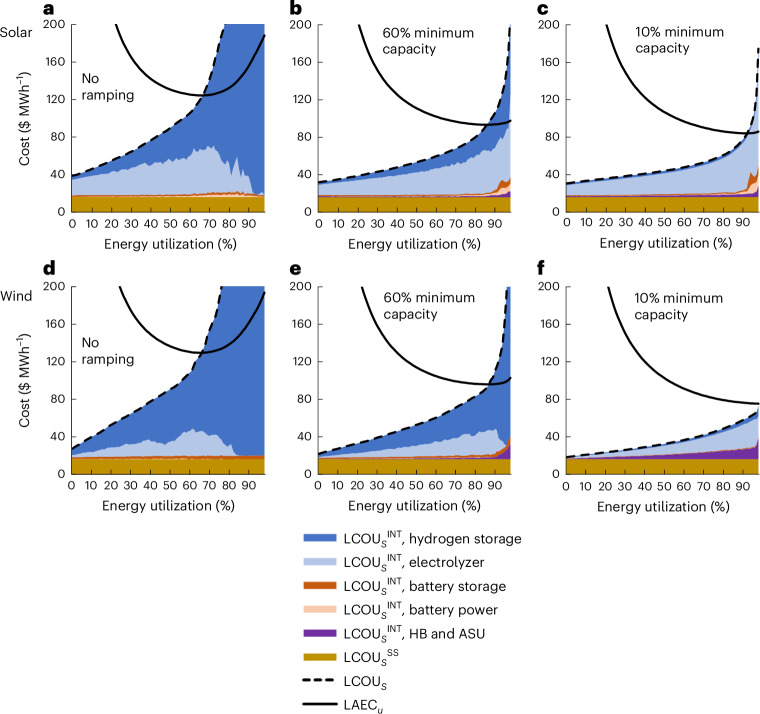
Energy utilization cost and total cost as a function of percent utilization at the best locations when ramping the HB process. The best locations are defined as having the lowest 10% of the LCOA without ramping, as that in Fig. 2. **a**–**f**, Results are averaged for each analysis with either no ramping (**a** and **d**), ramping down to 60% (**b** and **e**) or 10% (**c** and **f**) minimum operating capacity. The LCOU_*s*_^INT^, LCOU_*s*_^SS^ and LAEC_*u*_ values are determined as that in Fig. 2, with the LCOU_*s*_^INT^ value for the HB process and the ASU representing the additional capacity required due to ramping the HB process and ASU. Source data

The trend of decreasing LCOU_*u*__=opt_ with decreased minimum operating capacity (Fig. 4c,d) is explained in Fig. 5. With 60% minimum operating capacity (Fig. 5b,e), the LCOU_*s*_^INT^ value associated with hydrogen storage is drastically reduced compared with the case of no ramping (Fig. 5a,d), resulting in lower levels of curtailment (that is, LCOU_*s*_ intersecting LAEC_*u*_ at higher utilization levels). Nevertheless, the LCOU_*s*_^INT^ value associated with oversized electrolyzers remains considerable, particularly in the case of solar energy, because it is unaffected by decreasing the minimum operating capacity of the process. This limitation impedes further reduction in the LCOU_*u*__=opt_ value for solar energy by increasing the flexibility of HB. As a result, with 10% minimum operating capacity (Fig. 5c), it remains optimal to curtail a small amount of energy to remove the sharp increase in LCOU_*s*_ at the highest ‘peaks’ of solar energy.

**Extended Data Fig. 5:**
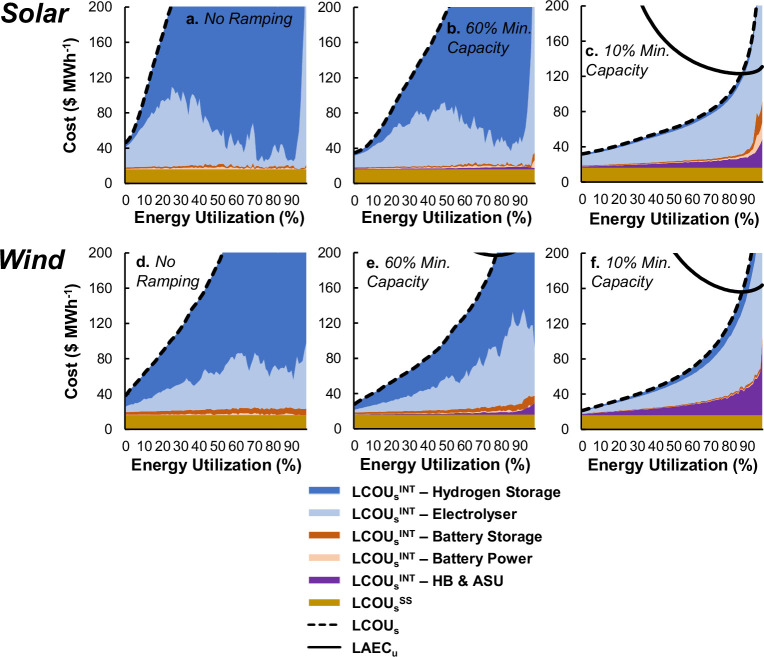
Energy utilization cost when ramping the HB process for locations with average solar or wind. The 10 sampled locations of solar or wind are analysed with either no ramping (**a** & **d**), ramping down to 60% minimum operating capacity (**b** & **e**) or down to 10% minimum operating capacity (**c** & **f**). The results for average solar or wind locations are the average of the 10 sampled locations. Source data

Conversely, wind energy relies more heavily on hydrogen storage and, therefore, achieves lower LCOU_*s*_ values as the minimum operating capacity decreases (Fig. 5e,f). With 10% minimum operating capacity, the HB process and air separation unit (ASU) contribute to the LCOU_*s*_^INT^ value because ramping down to low production leads to low fractional capacity and a notably oversized process. Despite this added cost, it is optimal to utilize 100% of the energy because the LAEC_*u*_ value does not intersect the LCOU_*s*_ value. Nevertheless, at the best wind energy locations, there is little possibility of further decreasing the LCOU_*s*_ value through increased flexibility because the LCOU_*s*_^INT^ value from hydrogen storage is negligible. This limit on the benefits of flexibility is also apparent for locations with average solar and wind energy (Extended Data Fig. 5), demonstrating that the electrolyzer cost will be a bottleneck for further economic improvements.

### Comparison with the cost and value of electricity on the grid

**Extended Data Fig. 6:**
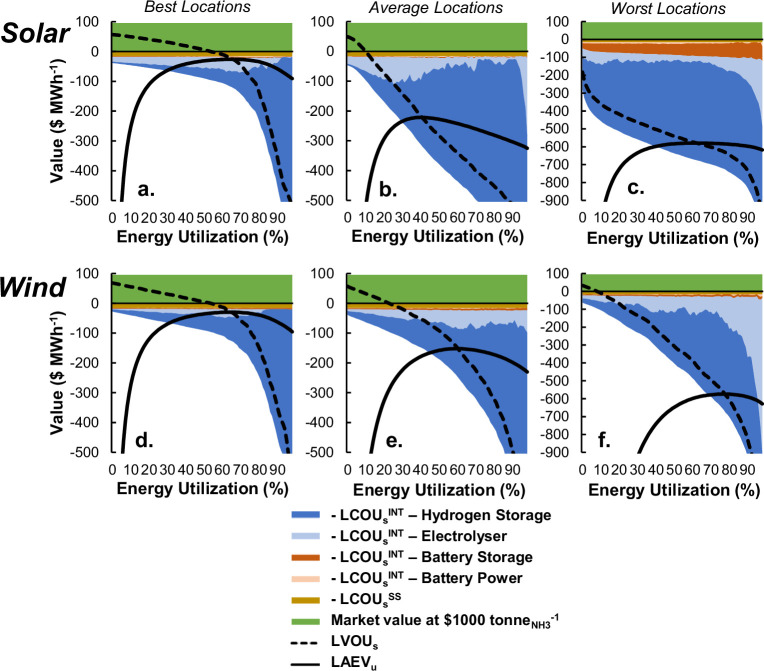
Value of utilizing energy to produce ammonia as a function of percent energy utilization. (**a**) the best solar locations (lowest 10% of LCOA). (**b**) average solar locations (45^th^–55^th^ percentile of LCOA). (**c**) worst solar locations (highest 10% of LCOA). (**d**) the best wind locations. (**e**) average wind locations. (**f**) worst wind locations. The results for 10 locations sampled in each category are averaged. The LCOU_s_ of each component is depicted as negative value because it is subtracted from the market value of energy in the form of ammonia, which is approximated to be $1000 t_NH3_^−1^. The LVOU_s_ is calculated as the sum of the market value and negatives of LCOU_s_. The Levelized Apparent Energy Value (LAEV_u_) is directly analogous to LAEC_u_ and is calculated by subtracting the LAEC_u_ from the market value of energy as ammonia. Source data

To provide a framework for understanding electrified chemicals production within the broader energy market, the LCOU is translated into the LVOU by subtracting the LCOU from the market value of energy sold as green ammonia (approximated as $1,000 t^−1^; ~$95 MWh^−1^) (equation (18)). The LVOU is similarly applied to strata (*s*) or total percent utilization (*u*) of energy, with the plots of LVOU_*s*_ (Extended Data Fig. 6) analogous to those of LCOU_*s*_ (Fig. 2).

**Fig. 6:**
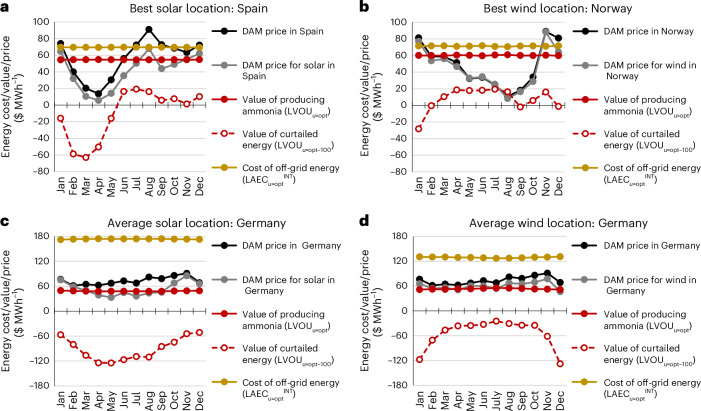
Cost and value of utilizing energy for ammonia production compared with electricity grid prices. **a**, Location in Spain (38.5° N, 1.5° W) in the lowest 10% of the LCOA with solar. **b**, Location in Norway (62.5° N, 10.5° E; bidding zone 1) in the lowest 10% of the LCOA with wind. **c**, Location in Germany (52° N, 11° E) in the 45th–55th percentile of the LCOA with solar. **d**, Location in Germany (52° N, 10° E) in the 45th–55th percentile of the LCOA with wind. The average hourly DAM price is from the most recent calendar year (Oct 2023 to Nov 2024)^50^. The price of solar or wind energy on the DAM is calculated by weighting the DAM prices by the amount of solar or wind energy produced at any time. The value generated by utilizing energy to produce ammonia (LVOU_*u*__=opt_), the value of curtailed energy (LVOU_*s*__=opt→100_) and the cost of producing off-grid energy (LAEC_*u*__=opt_^INT^) are shown with respect to each month averaged over the six years analyzed. Source data

A comparison of the LVOU_*u*_ for ammonia production with the prices of electricity on the grid determines the greatest economic potential for solar panels or wind turbines at a location. Similarly, the LAEC_*u*_^INT^ captures the apparent cost of intermittent supply of off-grid solar/wind energy compared with the cost of electricity on the local grid. Figure 6 presents the value of energy utilized to produce ammonia (LVOU_*u*__=opt_), the value of curtailed energy (LVOU_*s*__=opt→100_) and the cost of producing off-grid energy (LAEC_*u*__=opt_^INT^) for the case of ammonia production capable of ramping to 40% of the design capacity at select locations. Comparison is made with the monthly dependence of prices on the local grid, which is approximated from the average day-ahead market (DAM) price over the most recent calendar year (November 2023 to October 2024) as obtained from the ENTSOE-E Transparency Platform^50^. To describe the DAM price of solar/wind energy on each grid, the DAM price is weighted by the hourly production of solar/wind energy on the grid.

For both solar energy in Spain (Fig. 6a) and wind energy in Norway (Fig. 6b), the LVOU_*u*__=opt_ (solid red curve) is greater than the DAM price of solar or wind energy on the grid (gray curve). These countries have high penetrations of non-fossil power generators, with solar and wind energy noteworthy in Spain (46% solar and wind energy; 81% non-fossil energy) and hydropower prevalent in Norway (95% hydropower; 99% non-fossil energy), leading to low DAM prices, which fluctuate over the course of a year. In this circumstance, it is preferable to utilize solar/wind energy to produce green ammonia rather than selling to the grid. Conversely, LVOU_*u*__=opt_ does not depend on the month because curtailment removes the strata of energy with seasonal fluctuations. Therefore, utilizing renewable energy to produce green ammonia will be an economically beneficial market for renewable energy as the penetration of renewables continues to depress grid prices.

Unlike the value of energy utilized to produce ammonia, the value of curtailed energy (LVOU_*s*__=opt→100_) has seasonal dependence (Fig. 6, dashed red curves) as would be expected based on an excess of solar or wind energy in the summer or winter, respectively. Although energy curtailed from ammonia production could be sold on the grid, it is crucial to note that the value of curtailed solar energy in Spain follows similar monthly dependence as the price on the grid (Fig. 6a), demonstrating that both markets become saturated with solar energy at the same time. Therefore, as the penetration of renewable energy depresses grid prices further, there is a need for sector coupling to valorize curtailed energy in other off-grid applications. Curtailed wind energy in Norway, on the other hand, is the most plentiful (that is, the lowest value) when the grid price is the highest, indicating economic benefit from selling curtailed energy to the grid.

When considering locations of average solar or wind energy in Germany (Fig. 6c,d, respectively), the LVOU_*u*__=opt_ value does not definitively outperform the price of solar/wind energy on the grid (Fig. 6c,d). The LVOU_*u*__=opt_ value exceeds the price of solar or wind energy on the grid during only a few months due to depressed prices. Overall, the price on the grid across the year is higher than the value of producing ammonia because the penetration of non-fossil power generators is lower in Germany (providing 54% of energy) than in Spain or Norway, even though wind power provides ~35% of energy in Germany.

The cost of off-grid solar/wind energy described by LAEC_*u*__=opt_^INT^ demonstrates particularly different costs based on location. Solar energy in Spain and wind energy in Norway (Fig. 6a,b) have off-grid costs similar to the maximum price on the DAM, whereas solar and wind energy in Germany (Fig. 6c,d) exhibit off-grid costs of $50–$100 MWh^−1^ higher than grid prices. However, it is important to note that the disparity between the cost of off-grid and on-grid electricity does not suggest green ammonia is best produced using grid electricity, but rather sizeable carbon taxes or green subsidies are required to achieve parity in cost. Given a carbon intensity of ~400 g kWh^−1^ on Germany’s grid^51^, a carbon tax of $250 t_CO2_^−1^ is required to compensate for a $100 MWh^−1^ cost difference between off-grid and on-grid electricity. This degree of carbon taxation is beyond the range of currently planned carbon taxes in Europe, and therefore, the imprudent integration of green ammonia production with the grid is likely to hamper decarbonization goals because the true cost of off-grid energy in many locations is not reflected in current schemes of carbon accounting.

## Conclusions

Optimizing green ammonia production using solar and wind energy profiles at locations across the whole geography of Europe demonstrates that minimizing cost is decoupled from maximizing energy utilization when optimizing a chemical process with intermittent energy supply. In many locations, it is optimal to utilize less than 50% of the energy because it has a high cost to utilize the energy (LCOU), in many cases exceeding $500 MWh^−1^. In particular, the cost of utilizing energy in the highest portions of the energy profile is dominated by the cost of hydrogen storage because the energy is unevenly distributed throughout the year. When comparing the utilization costs stemming from the energy intermittency to the costs of utilizing steady-steady energy supply, it is clear that minimizing the overall cost is disconnected from maximizing the energy utilization, unlike fossil-powered ammonia production, which lacks utilization costs stemming from energy intermittency.

Combining solar and wind energy or ramping the production level of the HB process effectively reduces the LCOU, thereby decreasing the LCOA and optimal curtailment. For locations with an average supply of solar or wind energy, using the energy supplies in combination is particularly beneficial. Ramping the HB process similarly decreases the LCOU arising from hydrogen storage, but its impact is limited by the LCOU arising from electrolyzers, which is unaffected by ramping the HB process. Curtailment remains pervasive in both cases, but full energy utilization can be achieved when ramping to 10% minimum operating capacity at some of the best wind energy locations.

The value generated by utilizing solar/wind energy for ammonia production compared with the price of solar/wind energy on electricity grids depends on the composition of the grid. At locations in which grid electricity is primarily provisioned from non-fossil generators with low operating costs (for example, Spain or Norway), utilizing solar/wind energy for ammonia production is economically beneficial, whereas selling to the grid provides greater profit at locations more reliant on fossil-fuel power generation (for example, Germany). This demonstrates that the utilization of solar/wind energy for green chemicals production will become increasingly crucial for the economic build-out of renewables as their penetration on electricity grids continues to depress grid prices. Furthermore, by framing the economics of electrified chemicals production in terms of the cost to utilize energy and the value generated by utilizing energy, this work establishes a connection between the economics of electrified chemicals production and broader energy systems.

## Methods

### System design

In this study, an ammonia production system driven exclusively by renewable energy (solar/wind) is modeled using the key cost components for the process (Extended Data Fig. 1). Ammonia is produced at a normally constant rate of 230 t day^−1^, which corresponds to an energy consumption of 100 MW. Although this production capacity is less than conventional ammonia production (thousands of tons per day), it is anticipated that large-scale green ammonia production facilities will have smaller capacity due to less favorable economies of scale. Hourly profiles for solar and wind energy (acquired from the Photovoltaic Geographic Information System^52^ and New European Wind Atlas^53^, respectively) are considered for thousands of locations across Europe between the latitudes of 33.5° N–65° N and longitudes of 10° W–40° E in steps of 0.5°. In the case of wind energy, every location is categorized as onshore, offshore (ocean depth up to 50 m) or floating (ocean depth up to 1,000 m).

The energy supply is split into hydrogen production using proton exchange membrane electrolysis, nitrogen production through an ASU using pressure swing adsorption, and electrical power for the compressors and refrigeration of the HB loop. Hydrogen production and compression accounts for ~95% of the energy required to produce ammonia (36 GJ t_NH3_^−1^), whereas nitrogen production and electric power to the HB loop account for ~4% (1.3 GJ t_NH3_^−1^) and ~1% (0.3 GJ t_NH3_^−1^), respectively (Supplementary Table 3). Hydrogen production is assumed to be fully flexible such that it can follow the hourly resolution of solar/wind energy profiles and includes the costs of compression to 300 bar for high-pressure storage in a tank and/or feeding to the HB process at 100 bar. Although the flexible operation of electrolyzers could cause cell degradation and reduced performance, this problem arises primarily at the sub-hourly scale due to sudden changes in current and is, therefore, outside the scope of the hourly model in this study^54,55^. Further, even though electrolyzers have a minimum load for operation, this is removed from the model for computational efficiency and because the typical minimum load (5–10%) is small enough to have a negligible effect on the results^33^.

The temporally dependent allocation of power to hydrogen generation, nitrogen generation and electricity and the provision of electricity backup power in batteries (Li ion) are important considerations for process scheduling. When including the optimization of these variables in the overall process design (Supplementary Fig. 1 and Supplementary Information), the optimal configuration is nitrogen generation sized at approximately the same capacity as the HB process, minimal nitrogen storage, and battery backup power for nitrogen generation and electricity for the HB process. This configuration is optimal due to the relative capital costs of storing energy ($16,000 MWh_H2_^−1^, $72,000 MWh_N2_^−1^ and $165,000 MWh_Battery_^−1^, respectively) and the comparatively low cost of oversizing the power capacity of the battery bank such that recharging can be prioritized whenever energy is available, thereby decreasing the storage capacity of the batteries. Given this optimal allocation of power, a partially simplified model (Extended Data Fig. 1) is implemented, which allows optimal power allocation to the electrolyzer and battery bank, while setting the nitrogen generation to the capacity of the HB process and removing nitrogen gas storage. This simplified model facilitates faster optimization and results in negligible change in process design and economics (Supplementary Table 1).

### Economic optimization

The optimization of green ammonia production consists of a linear programming problem in MATLAB (v.9.13.0) (ref. ^56^), which uses a dual-simplex algorithm to solve for (1) the capacity of solar panels and/or wind turbines; (2) the capacity of hydrogen production and battery power; (3) the capacity of hydrogen and battery storage; and (4) the scheduling of hydrogen and battery power for every hour over the course of a year, assuming complete foresight. The objective of optimization is to minimize the LCOA for a particular location (*l*) and year (*y*), defined as the total annualized capital and operating costs in US dollars of all the equipment normalized by the yearly production of ammonia (*P*: 84,000 t), as shown in equation (1):

1 $${{{\rm{LCOA}}}}_{l,y}\,\left[{{\rm{US}}}{{\$\,t_{\rm{NH}3}^{-1}}}\right]=\mathop{\min }\limits_{{C}_{k,{{\rm{CAPEX}}}},{C}_{k,{{\rm{OPEX}}}}}\left(\frac{\,\sum _{k}A \times {C}_{k,{{\rm{CAPEX}}}}\left({X}_{k}\right)+\sum _{k}{C}_{k,{{\rm{OPEX}}}}\left({X}_{k}\right)}{P}\right),$$

where *C*_*k*,CAPEX_ and *C*_*k*,OPEX_ are the capital cost and operating cost, respectively, of the process component (*k*) as a function of installed capacity (*X*), and *A* is the annualization factor for a 25-year process lifetime to payback capital investment and a 7% discount factor (that is, interest rate used in the discounted cash flow), which are typical parameters for optimizing green ammonia production^18,25^. The objective in equation (1) is constrained by equations (2)–(13):

2 $$0\le \mathop{\sum }\limits_{t}^{T}{{{\rm{su}}}}_{t} \times {X}_{{{\rm{SU}}}}-D,$$

3 $$0\le {h}_{t}+{e}_{t}\le \left({{{\rm{su}}}}_{t} \times {X}_{{{\rm{SU}}}}\right),\,\forall t$$

4 $${0\le h}_{t}\le {X}_{{\rm{H}}},\,\forall t,$$

5 $$0\le {e}_{t}-{D}_{{{\rm{HB}}}+{{\rm{ASU}}}}\le {X}_{{{\rm{BP}}}},\,\forall t,$$

6 $$0\le {{{\rm{hs}}}}_{t}\le {X}_{{{\rm{HS}}}},\,\forall t,$$

7 $$0\le {{{\rm{bs}}}}_{t}\le {X}_{{{\rm{BS}}}},\,\forall t,$$

8 $${{{\rm{bs}}}}_{t}={{{\rm{bs}}}}_{t-1}+{e}_{t}-{D}_{{{\rm{HB}}}+{{\rm{ASU}}}},\,\forall t > 1,$$

9 $${{{\rm{hs}}}}_{t}={{{\rm{hs}}}}_{t-1}+{h}_{t}\times Y-{D}_{{\rm{H2}}},\,\forall t > 1,$$

10 $${{{\rm{bs}}}}_{t}={{{\rm{bs}}}}_{0}+{e}_{t}-{D}_{{{\rm{HB}}}+{{\rm{ASU}}}},\,t=1,$$

11 $${{{\rm{hs}}}}_{t}={{{\rm{hs}}}}_{0}+{h}_{t}\times Y-{D}_{{\rm{H2}}},\,t=1,$$

12 $$0\le {{{\rm{bs}}}}_{0}={{{\rm{bs}}}}_{t}\le {X}_{{{\rm{BS}}}},\,t=8,760,$$

13 $$0\le {{{\rm{hs}}}}_{0}={{{\rm{hs}}}}_{t}\le {X}_{{{\rm{HS}}}},\,t=8,760,$$

where su_*t*_, *h*_*t*_, *e*_*t*_, hs_*t*_ and bs_*t*_ are the energy supply (MW MWp^−1^), energy allocated for hydrogen production (MW), energy allocated to electricity (MW), hydrogen gas storage inventory and battery electricity storage inventory at hour *t*, respectively, with bs_0_ and hs_0_ being the initial inventory states. *X*_SU_, *X*_H_, *X*_BP_, *X*_HS_ and *X*_BS_ are the capacity of solar panels/wind turbines (MWp), hydrogen generation (MW), battery power (MW; assumed to be determined by recharging power), hydrogen storage (kg) and battery storage (kWh), respectively. *D*, *D*_HB+ASU_ and *D*_H2_ are the total energy demand for ammonia production (100 MW), the electricity demand for nitrogen generation and the HB process (MW) and the hydrogen demand for ammonia production (kg h^−1^). *Y* is the conversion factor of electricity to hydrogen (kg MWh^−1^) based on the electrolyzer efficiency and hydrogen compression, which is assumed to be independent of the production rate. In this model, it is approximated that there are no energy losses when storing electricity in a battery.

By formulating optimization with energy utilization at each hour as independent variables (equation (3)) rather than relying on a procedure for allocating power over a simulated year of energy supply^15,42^, this model captures the use of energy curtailment for both (1) reducing the power intake capacity of the system (*X*_H_ and *X*_BP_) and (2) reducing the storage capacity of the system (*X*_HS_ and *X*_BS_). When the optimization is constrained to achieve full energy utilization (no curtailment), equation (3) is an equality as opposed to an inequality. Optimization was conducted for each of the six years of solar/wind energy data from 2013 to 2018, with the results for all years averaged to generate the approximate LCOA for a location using solar/wind energy. Although a more thorough calculation of LCOA at a particular location should account for longer variations in solar/wind energy^57^, data for six years have been used in this analysis to facilitate investigating the economic trends at many locations and illustrate a framework for understanding the relative economic drivers. A detailed summary of the capital and operating costs as well as the energy requirements are provided in Supplementary Tables 2 and 3. It should be noted that these parameters are estimates intended to demonstrate the relationship between LCOA, energy curtailment and the cost of energy utilization as opposed to prescribing a precise cost of ammonia at any particular location.

When analyzing a combination of solar and wind at a location, the optimization solves for the installed capacity of both solar panels and wind turbines at onshore locations (offshore solar is not considered in the model), with energy supply and capacity terms for both solar and wind appearing in equations (1)–(3) in this case. When analyzing the effect of ramping the HB process (including ASU)^14^, the optimization also solves for the installed capacity of the HB process and the production scheduling of the HB process during every hour of the year. As a result, the demand for ammonia production becomes a time-dependent variable with constraints on its range (that is, minimum capacity) and change between consecutive time points (that is, rate of ramping). To partially simplify the model, it is assumed that the HB + ASU units would only change the production level on a four-hourly rather than hourly basis (Supplementary Information), and the energy consumption of the HB + ASU unit scales proportionally with the production rate. In all the analyses, the optimization is constrained to produce the same absolute amount of ammonia per year (84,000 t).

### Apparent cost of energy

To investigate the economic drivers for curtailment as they relate to location and process configuration, the apparent cost of energy in a profile of solar/wind power is evaluated from the perspective of costs associated with the ammonia production process. As shown conceptually in Extended Data Fig. 3, the apparent energy cost determines the amount of energy curtailed because it reflects how much it costs to utilize the energy. The analysis of apparent cost is conducted by calculating the change in cost as a function of the percent of energy utilized (*u*) for a given capacity of solar panels or wind turbines (Extended Data Fig. 2). One should note that the percent of energy curtailed is the difference between 100% and percent of energy utilized. As energy utilization increases from 0% to 100%, the amount of energy used increases linearly. Similarly, the cost *C*_*k*_ of process component *k* increases according to the change in capacity of the process component needed to utilize such energy. Conversely, the cost of energy supply per unit of energy used (*C*_E_; $ MWh^−1^) decreases from an asymptote at 0% until it reaches the cost to produce energy using solar panels or wind turbines at 100% utilization, typically referred to as the levelized cost of electricity from solar panels or wind turbines. Using the framework illustrated in Extended Data Fig. 3, the LCOU for energy up to percent utilization *u* can be defined using equation (14):

14 $${{{\rm{LCOU}}}}_{u}\,\left[{{\rm{US}}}\$\,{\rm{MWh}}^{-1}\right]=\frac{\sum _{k}{C}_{k,u}}{{E}_{u}},$$

where LCOU_*u*_ is the summation of the cost of each process component (which includes both annualized capital costs and operating costs in US dollars) over the energy utilized (*E*_*u*_) on the basis of one year. To analyze how cost changes with energy utilized, the LCOU value is also defined with respect to a stratum (*s*) of utilized energy by taking the derivative cost with energy:

15 $${{{\rm{LCOU}}}}_{s}\,\left[{{\rm{US}}}\$\,{\rm{MWh}}^{-1}\right]=\frac{{\rm{d}}\left(\sum _{k}{C}_{k}\right)}{{{\rm{d}}E}}=\frac{\sum _{k}\left({C}_{k,u}-{C}_{k,u-1}\right)}{{E}_{u}-{E}_{u-1}}=\frac{\sum _{k}\left({C}_{k,u}-{C}_{k,u-1}\right)}{\Delta E},$$

where the change in utilized energy and cost of utilization are found using discrete steps of percent utilization (Δ*u*), which correspond to discrete steps in utilized energy (Δ*E*). The relationship between LCOU_*u*_ and LCOU_*s*_ is then given by equation (16):

16 $${{{\rm{LCOU}}}}_{u}=\frac{1}{{E}_{u}}{\int }_{0}^{{{E}_{u}}}{{{\rm{LCOU}}}}_{s}{{\rm{d}}E}=\frac{1}{u\times \Delta E}\mathop{\sum }\limits_{s=1}^{u}{{{\rm{LCOU}}}}_{s}\Delta E=\frac{1}{u}\mathop{\sum }\limits_{s=1}^{u}{{{\rm{LCOU}}}}_{s},$$

where the total cost (LCOU_*u*_) is calculated from the integral of LCOU_*s*_ over the amount of energy utilized. Since the energy steps are discrete (Δ*E*), the LCOU_*u*_ value simply becomes the average of the LCOU_*s*_ values over strata 1 to *u*. Using the LCOU_*u*_ value, the total cost of energy at percent utilization *u* is defined as LAEC_*u*_:

17 $${{{\rm{LAEC}}}}_{u}\,\left[{{\rm{US}}}\$\,{\rm{MWh}}^{-1}\right]={C}_{E,u}+{{{\rm{LCOU}}}}_{u}=\frac{{C}_{{{\rm{panels}}}/{{\rm{turbines}}}}}{{E}_{u}}+\frac{1}{u}\mathop{\sum }\limits_{s=1}^{u}{{{\rm{LCOU}}}}_{s},$$

where the cost of energy supply (*C*_E,*u*_) is the total cost of solar panels or wind turbines on an annual basis (*C*_panel/turbines_) divided by the total energy utilized (*E*_*u*_). It is important to note that to calculate LCOU and LAEC, the cost of each process component at a given percent utilization is determined by optimizing the overall process design and setting energy utilization as a constraint.

To further highlight the effect of different process components on cost, the LCOU is classified according to which components are considered in equations (14)–(17). When considering the capacity of electrolyzers, ASU and HB process required to utilize energy at steady state (that is, energy supply is constant), the cost is termed LCOU_*s*_^SS^. This cost scales linearly (that is, LCOU_*s*_^SS^ is constant) because the capacity of the components is proportional to the energy utilized. When considering the additional capacity of electrolyzers, hydrogen storage, battery power (MW) and battery storage (kWh) required to utilize energy considering its intermittency, the cost is termed LCOU^INT^. This cost does not scale linearly with percent utilization because LCOU_*s*_^INT^ is dependent on the temporal distribution of energy in stratum *s*. Similarly, the LAEC_*u*_ value can be classified according to whether it considers steady-state components (LAEC_*u*_^SS^) or intermittent components (LAEC_*u*_^INT^).

In some cases, it is also beneficial to delineate the cost of utilizing energy from different energy supplies when they are used in combination (that is, solar and wind energy), or the cost for different months of the year to investigate temporal patterns. This analysis of cost is accomplished by distributing the strata of energy to each of the energy supplies or months according to their relative energy utilization, and the algorithm for executing this procedure is described in the Supplementary Information.

The cost of utilizing energy is converted into the value generated through energy utilization by subtracting the cost of utilizing energy from the market value (MV) of the energy when it is sold into market *m*:

18 $${{\rm{LVOU}}}\,\left[{{\rm{US}}}\$\,{\rm{MWh}}^{-1}\right]={{{\rm{MV}}}}_{m}-{{\rm{LCOU}}},$$

where LVOU is the levelized value of utilizing energy. The LVOU value can be with respect to percent utilization (*u*) or stratum (*s*) when using LCOU_*u*_ or LCOU_*s*_, respectively. In the case of green ammonia production, renewable energy is sold into the green ammonia market (MV_gNH3_), which is assumed to be priced at ~$1,000 t_NH3_^−1^, translating into a market value of ~$95 MWh^−1^ based on the energy input. Although a market for green ammonia does not currently exist and this price for green ammonia is higher than the historical price of ammonia ($400–700 t_NH3_^−1^), it is used here to illustrate the comparison with grid prices.

## Supplementary information

Supplementary Information Supplementary Figs. 1–3, Tables 1–5 and detailed methodology.

## Source data

Source Data Fig. 1 Statistical source data.

Source Data Fig. 2 Statistical source data.

Source Data Fig. 3 Statistical source data.

Source Data Fig. 4 Statistical source data.

Source Data Fig. 5 Statistical source data.

Source Data Fig. 6 Statistical source data.

Source Data Extended Data Fig./Table 2 Statistical source data.

Source Data Extended Data Fig./Table 4 Statistical source data.

Source Data Extended Data Fig./Table 5 Statistical source data.

Source Data Extended Data Fig./Table 6 Statistical source data.

## Data Availability

The datasets presented in this work are available at Apollo, the University of Cambridge data repository, which can be accessed at 10.17863/CAM.111758 (ref. ^58^).

## Code availability

The codes implemented in this work are available at Apollo, the University of Cambridge data repository, which can be accessed at 10.17863/CAM.111760 (ref. ^59^), and also available via GitHub at https://github.com/cjs227/CostEffvsEnUt (ref. ^60^).
