# Supplementary material for: Cost efficiency versus energy utilization in green ammonia production from intermittent renewable energy
Source: Nat Chem Eng. 2025 Apr 18;2(4):261–72. doi: 10.1038/s44286-025-00207-9 (PMC12018267; doi:10.1038/s44286-025-00207-9)
Supplement: Supplementary file 1 — Supplementary Figs. 1–3, Tables 1–5 and detailed methodology. [file 44286_2025_207_MOESM1_ESM.pdf]

nature chemical engineering

Supplementary information https://doi.org/10.1038/s44286-025-00207-9

Cost efficiency versus energy utilization in green ammonia production from intermittent renewable energy

In the format provided by the authors and unedited

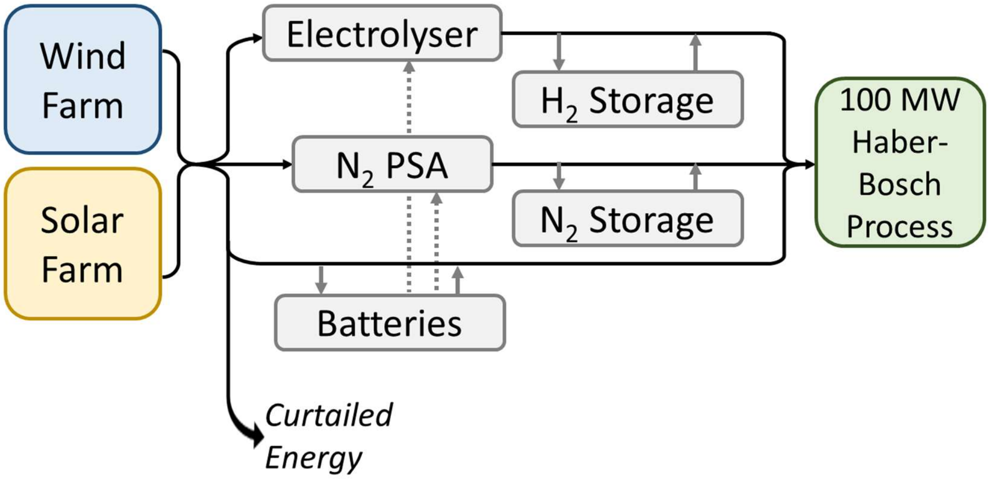

Figure S.1 | Schematic of full-scope model for ammonia production with degrees of freedom associated with the temporal allocation of each process stream. The supply of energy is optimally allocated to hydrogen production, nitrogen production, electricity for the HB process or curtailed. The capacity of hydrogen storage (pressurized tanks), nitrogen storage (pressurized tanks) and batteries (li-ion) is similarly optimized to minimize cost. The batteries can also be utilized to power the hydrogen or nitrogen production if it is optimal. The optimization results for this system as compared to the simplified system used for analysis (Figure 1) are shown in Table S.1.

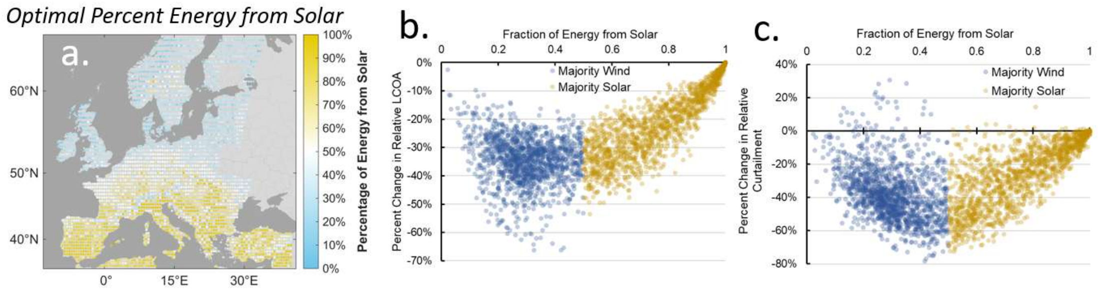

Figure S.2 | Optimization of solar and wind with curtailment. (a) Map of the fraction of total energy sourced from solar energy. (b) The change in the optimal LCOA as a function of fraction of energy sourced from solar. (c) The change in optimal curtailment as a function of fraction of energy sourced from solar.

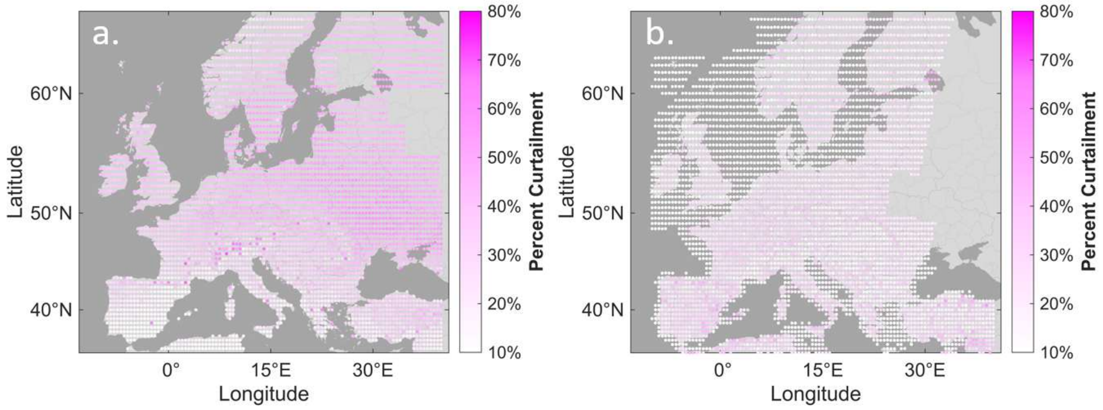

Figure S.3 | Optimal curtailment with ramping of the HB process down to 60% of the installed capacity. a & b. percent curtailment for solar (a) and wind (b) energy with optimal ramping of the HB process.

Table S.1 | Simplified model optimized cost of ammonia (LCOA) compared to full-scope model.

Location (Latitude_Longitude)	LCOA full-scope model ($/tonne)	LCOA simplified Model ($/tonne)	LCOA Percent Difference
**Good Solar**			
38°N_2.5°W	1208	1211	0.21
37.5°N _36°E	1495	1497	0.13
36°N _40°E	1343	1347	0.24
37.5°N _7.5°W	1242	1245	0.24
38.5°N _1.5°W	1160	1163	0.20
38.5°N _8°W	1318	1322	0.26
37°N _6°W	1171	1174	0.25
37°N _3.5°W	1177	1180	0.29
42°N _2.5°E	1410	1413	0.22
35.5°N _1.5°E	1300	1303	0.21
**Average Solar**			
53°N _2.5°W	2980	3014	1.11
53°N _6°E	3083	3106	0.74
52.5°N _7°W	2965	2999	1.15
47°N _39°E	3220	3223	0.11
48.5°N _35°E	3023	3028	0.14
49°N _6.5°E	2899	2907	0.29
51.5°N _19°E	3130	3139	0.27
47.5°N _32°E	3165	3169	0.13
52°N _11°E	2892	2912	0.71
47°N _6°E	3359	3496	4.08
**Poor Solar**			
64.5°N _13.5°E	6751	7237	7.19
64°N _10.5°E	6901	7588	9.95
64.5°N _33.5°E	6169	6465	4.81
64.5°N _27.5°E	6087	6384	4.88
65°N _19.5°E	6359	6882	8.23
65°N _18.5°E	6323	6853	8.38
63°N _12.5°E	6696	7026	4.94
64.5°N _17.5°E	6316	6789	7.48
62°N _7°W	6412	6549	2.13
59°N _6.5°E	7538	7657	1.59
**Good Wind**			

58.0045°N _4.999°E	1446	1450	0.33
56.4953°N _5.4933°E	1456	1462	0.39
56.5008°N _5.0235°W	1216	1220	0.32
61.0028°N _9.5175°W	1479	1487	0.48
57.4895°N _5.0206°W	865	870	0.50
57.5073°N _5.5015°W	998	1002	0.40
59.505°N _5.4802°W	1427	1432	0.38
58.5113°N _8.9832°W	1383	1389	0.47
56.9913°N _5.4859°E	1444	1450	0.42
53.494°N _10.0107°W	954	956	0.17
**Average Wind**			
41.4964°N _17.0096°E	2755	2768	0.47
60.5094°N _25.5258°E	2962	2980	0.62
34.7352°N _10.486°E	2149	2151	0.09
48.5027°N _2.0189°W	2203	2213	0.46
39.9951°N _7.5117°W	2666	2676	0.36
64.5069°N _23.9739°E	2122	2126	0.18
53.5124°N _18.491°E	2698	2706	0.28
48.9954°N _30.4876°E	2654	2662	0.31
48.0116°N _22.0206°E	3019	3036	0.56
35.4885°N _13.5125°E	2895	2909	0.48
**Poor Wind**			
45.5077°N _15°E	5246	5264	0.33
36.5117°N _31.9902°E	12312	12334	0.18
37.0085°N _34.5053°E	6937	6946	0.13
43.9991°N _17.4855°E	5074	5081	0.14
37.5022°N _5.9935°W	5309	5329	0.37
46.9939°N _9.9999°E	6979	6984	0.08
46.9939°N _9.9999°E	6979	6984	0.08
45.4899°N _8.0156°E	11142	11175	0.29
38.0062°N _40.0164°E	6404	6426	0.33
45.011°N _9.5019°E	6971	7005	0.48

Table S.2 | Economic Parameter Estimations

Parameter	Value	Notes	Relevant Sources**
Panel capex*	0.47 M€ MWp^{-1}		1
Onshore Turbine capex*	1.12 M€ MWp^{-1}		2
Offshore Turbine capex*	1.87 M€ MWp^{-1}	Distance from shore not included in the cost estimation.	3
Floating Turbine capex*	3 M€ MWp^{-1}	Distance from shore not included in the cost estimation. Production would most likely be location as sub-stations offshore.	4,5
Electrolyser capex	700 \$ kW^{-1}	The electrolyser is assumed to be PEM due to superior flexibility compared to alkaline, but alkaline could also achieve similar flexibility with modular, independently operated stacks. The capex used in this study reflects the optimistic cost including expected reductions in the case of PEM, but is the readily available cost in the case of alkaline.	6-8
ASU capex	50 k\$ $(t_{NH3}\ day^{-1})^{-1}$		9-13
H_2 compressor capex	60 k\$ $(t_{NH3}\ day^{-1})^{-1}$	See Haber Bosch capex notes. 50% of capex attributed to compressors is approximated to be for H_2. While 3X as much hydrogen is compressed compared to N_2, the starting pressure is 30 bar from the electrolyser rather than ~ 1 bar from the ASU.	11,14
N_2 compressor capex	60 k\$ $(t_{NH3}\ day^{-1})^{-1}$	See H_2 compressor notes.	
H_2 storage capex*	900 \$ kg^{-1}	Calculated from a base cost of ~500 € kg^{-1}, adjust considering that only ~2/3 of the gas storage is available for us in a Haber Bosch process operated at a minimum of 100 bar.	9,13,15
N_2 storage capex*	50 \$ kg^{-1}	Approximated to be half the cost of hydrogen on a volume basis due to lack of hydrogen embrittlement and permeation.	
Battery storage capex*	150 € kWh		16
Battery power capex*	270 € kW		16
Haber Bosch capex	125 k\$ $(t_{NH3}\ day^{-1})^{-1}$	This represents only the HB loop. It was approximated using a value of 250 k\$ $tNH3^{-1}$ day^{-1} for the whole process, with approximately 50% of the cost attributed to the compressors which were sized independently. The overall	9-11,14,17-21

		cost was approximated as within the range reported in literature. (50 – 900 k$ tNH3^{-1} day^{-1})	
Panel O&M*	11300 € MW^{-1} yr^{-1}		1
Onshore Turbine O&M*	14000 € MW^{-1} yr^{-1}		2
Offshore Turbine O&M*	50000 € MW^{-1} yr^{-1}		3
Floating Turbine O&M	25% of total cost	Applied on an annual basis relative to the annualized capital cost.	4,5
Electrolyser O&M<br>ASU O&M<br>Compressor O&M<br>Haber Bosch O&M	2% of total capital cost	This is generally considered to be a reasonable approximation of the O&M costs for most process equipment.	8-10,12,17,22,23

* Euros converted to US dollars using a conversion of 1.1 $ €$^{-1}$.

** The Relevant Sources provide the references which inform the determination of the cost parameter. When multiple sources are present, the cost parameter is approximated from that presented in the sources.

Table S.3 | Energy Consumption Parameters

Parameter	Value	Notes	Relevant Sources
Electrolyser	55.5 kWh kg_{H2}^{-1}	60% of H_2 LHV of 33.3 kWh kg_{H2}^{-1}	[8,24]
ASU	0.25 kWh kg_{N2}^{-1}		[9,13,17,21,25]
Hydrogen compressor	13 kJ mol_{NH3}^{-1}		[14,21]
Nitrogen compressor	10 kJ mol_{NH3}^{-1}		[14,21]
Haber Bosch loop	5 kJ mol_{NH3}^{-1}		[14,21]

Note: the efficiency of the batteries is approximated as 100%.

Table S.4 | Nomenclature in optimization algorithm

Variable/ Parameter	*Description*	*Units*
LCOA	*Levelized cost of ammonia on a yearly basis*	*$ tonne-1*
$LCOU_s$	*Levelized cost of utilizing energy in stratum s of an energy profile*	$\$ MWh^{-1}$
LVOUs	*Levelized value generated by utilizing energy in stratum s of an energy profile*	$\$ MWh^{-1}$
$LAEC_u$	*Total cost to utilize energy up to utilization level u*	$\$ MWh^{-1}$
LAEV	*Total value generated by utilizing energy up to utilization level u*	$\$ MWh^{-1}$
A	*Capital cost annualization factor*	*NA*
$C_{k,CAPEX}$	*Capital cost of process component k*	*$*
$C_{k,OPEX}$	*Operating cost of process component k per year*	$\$ yr^{-1}$
C_k	*Total yearly capital and operating cost of component k*	$\$ yr^{-1}$
X_{SU}	*Installed capacity of energy supply (i.e. solar panels or wind turbines)*	*GWp*
X_H	*Installed capacity of hydrogen generation*	*GW*
X_{BP}	*Installed capacity of battery power*	*GW*
X_N	*Installed capacity of nitrogen generation (if applicable)*	*GW*
X_{HS}	*Installed capacity of hydrogen storage*	*Kg*
X_{BS}	*Installed capacity of battery storage*	*Kg*
X_{NS}	*Installed capacity of nitrogen storage (if applicable)*	*kWh*
X_{HB+ASU}	*Installed capacity of HB process and ASU (if applicable)*	*GW*
su_t	*Energy supply at time t (parameter based on solar or wind energy at the location)*	*GW*
h_t	*Hydrogen generation power at time t*	*GW*
e_t	*Electricity power usage at time t*	*GW*
n_t	*Nitrogen generation power at time t*	*GW*
hs_t	*Hydrogen storage inventory at time t*	*kg*
ns_t	*Nitrogen storage inventory at time t*	*kg*
bs_t	*Battery storage inventory at time t*	*kWh*
bh_t	*Battery power to hydrogen generation at time t (if applicable)*	*GW*
bn_t	*Battery power to nitrogen generation at time t (if applicable)*	*GW*
$d_{H2,\tau}$	*Demand for hydrogen in the HB process at time τ (if applicable)*	$Kg\ hr^{-1}$
$d_{HB+ASU,\tau}$	*Demand for power in the HB process and ASU at time τ (if applicable)*	*GW*
D_{H2}	*Demand for hydrogen in the HB process (parameter)*	$Kg\ hr^{-1}$
D_{HB+ASU}	*Demand for power in HB process and ASU (parameter)*	*GW*
Y	*Conversion of electrical power into hydrogen*	$Kg\ GWh^{-1}$
Z	*Ratio of electrical demand to hydrogen demand in the HB process*	$GW\ (kg\ s^{-1})^{-1}$

Table S.5 | Locations selected for each class of energy supply profile.

Top 10% Solar Locations	Average Solar Locations*	Bottom 10% Solar Locations
38°N – 2.5^{o}W	53°N – 2.5^{o}W	64.5^{o}N – 13.5^{o}E
37.5^{o}N – 36°E	53°N – 6°E	64°N – 10.5^{o}E
36°N – 40°E	52.5^{o}N - 7°W	64.5^{o}N – 33.5^{o}E
37.5^{o}N – 7.5^{o}W	47°N – 39°E	64.5^{o}N – 27.5^{o}E
38.5^{o}N – 1.5^{o}W	48.5^{o}N – 35°E	65°N – 19.5^{o}E
38.5^{o}N – 8°W	49°N – 6.5^{o}E	65°N – 18.5^{o}E
37°N – 6°W	51.5^{o}N – 19°E	63°N – 12.5^{o}E
37°N – 3.5^{o}W	47.5^{o}N – 32°E	64.5^{o}N – 17.5^{o}E
42°N – 2.5^{o}E	52°N – 11°E	62°N – 7°W
35.5^{o}N – 1.5^{o}E	47°N – 6°E	59°N – 6.5^{o}E
**Top 10% Wind Locations**	**Average Wind Locations***	**Bottom 10% Wind Locations**
52°N - 9°W	64°N – 12°E	37°N – 35°E
37°N – 26°E	61.5^{o}N – 26.5^{o}E	37°N – 28°E
55°N – 3°E	53°N – 13°E	44.5^{o}N – 11°E
55°N – 7°E	45°N – 7°E	46.5^{o}N – 13°E
57.5^{o}N – 10°E	52°N – 10°E	60°N – 10°E
62.5^{o}N – 10.5^{o}E	41.5^{o}N – 15.5^{o}E	37.5^{o}N – 38°E
62°N – 7.5^{o}E	50.5^{o}N – 19.5^{o}E	45°N – 1°E
56°N – 5°E	64.5^{o}N – 33°E	43°N – 19.5^{o}E
54.5^{o}N – 8°E	59.5^{o}N – 10.5^{o}E	35°N – 6°W
58.5^{o}N – 3.5^{o}W	42°N – 3°W	40.5^{o}N – 1.5^{o}E

*Average is defined as being between the 45th and 55th percentiles.

Table S.6 | Locations selected for each class of combined solar and wind profiles.

Top Solar and Wind*	Top Solar and Average Wind**	Top Wind and Average Solar**	Average Solar and Wind**
41.5^{o}N – 9°E	37°N – 11°E	51°N – 4.5^{o}W	52.5^{o}N – 18°E
37°N – 33°E	37°N – 2°W	39.5^{o}N – 40°E	49.5^{o}N – 22°E
36°N – 10.5^{o}E	40°N – 22°E	54°N – 1.5^{o}W	50°N – 24°E
40°N – 34°E	41.5^{o}N – 0.5^{o}W	47°N – 11.5^{o}E	51°N – 23°E
43.5^{o}N – 4.5^{o}E	36°N – 0.5^{o}E	45.5^{o}N – 14.5^{o}E	52°N – 8°E
37°N – 25.5^{o}E	38.5^{o}N – 33.5^{o}E	53.5^{o}N – 9.5^{o}W	53°N – 15.5^{o}E
36°N – 4.5^{o}E	37°N – 30.5^{o}E	57.5^{o}N – 2.5^{o}W	51.5^{o}N – 19°E
37°N – 4.5^{o}W	39°N – 17°E	52°N – 9°W	48.5^{o}N – 27.5^{o}E
38°N – 23.5^{o}E	40°N – 3.5^{o}W	54.5^{o}N – 2°W	48.5 – 28.5^{o}E
38.5^{o}N – 26.5^{o}E	40°N – 7.5^{o}W	50.5^{o}N – 4°W	45.5^{o}N - 3°E

*Top solar and wind locations are defined as within the 15th percentile for each because there are too few locations within the 10th percentile of each.

**Top locations are defined as within the 10th percentile. Average locations are defined as between the 45th and 55th percentile.

Detailed Methodology

i. Data collection and processing

Solar power data was acquired for 3577 locations across Europe in increments of half a degree latitude and longitude using the Photovoltaic Geographic Information System (PVGIS) provided by the European commission.[26] The power output (watts) per installed capacity of solar panels (kWp) was generated on an hourly basis for the years 2013 – 2018, assuming a crystalline silicon panel, 14% system losses and fixed mounting with slope and azimuth optimized by the PVGIS tool to maximize power outlet.

Wind speed data was acquired for 4553 locations across Europe in increments of half a degree latitude and longitude using the New European Wind Atlas, a free, web-based application developed, owned and operated by the NEWA Consortium. For additional information see www.neweuropeanwindatlas.eu.[27] Reference wind speeds at a height of 10 m were converted to the wind speed at a turbine hub height of 100 m or 130 m for onshore and offshore/floating wind, respectively, using a scaling exponent of 0.143.[28] The wind speed was converted to turbine power output using Equation 1[29]

$$P_{turbine}^{actual} = P_{turbine}^{rated}\left(\frac{v^3 - v_i^3}{v_r^3 - v_i^3}\right) \tag{S.1}$$

where P_{out} is the output power in watts, P_{rated} is the rated power in watts, v is the wind speed during a given hour, v_i is the cut in wind speed of the wind turbine (approximated to be 3 m s^{-1} [30]) and v_r is the rated wind speed (approximated to be 13 m s^{-1} [29]). At the cutout wind speed, which was approximated to be 25 and 31 m s^{-1} for onshore and offshore/floating wind, respectively, P_{out} drops to zero due to limitations in wind turbine stability.

The dataset of wind power at 4553 locations was divided into onshore wind, offshore wind to a depth of 50 m and floating wind to a depth of 1000 m. Locations with a water depth greater that 1000 m were excluded from the dataset. The elevation and water depth as a function of latitude and longitude was acquired from the Global Bathymetric Chart of the Oceans.[31]

ii. Economic parameters

The necessary economic and energy parameters utilized for each unit in the green ammonia production process (Figure 1) are summarized in Tables S.2 and S.3, respectively.

iii. Optimization algorithm

The relevant variables used in the optimization algorithm are listed in Table S.4. The optimization of the full-scope model (Figure S.1) has an identical objective function as the simplified model (Figure 1; Equation 1). Mainly, the levelized cost of producing ammonia is minimized over the annualized capital cost and operating costs of each process component, with the annualization factor being defined by Equation S.2

$$A = \left(\frac{R}{1-(1+R)^{-Y}}\right) \tag{S.2}$$

where R is the discount rate (7%) and Y is the lifetime in years (25). However, in the full-scope model, there are additional variables which are optimized using addition constraints, the full like of which is listed in Equations S.3 – S.21.

$$0 \le \sum_{t}^{T} su_t * X_{SU} - D \quad \text{(S.3)}$$

$$0 \le h_t + \ e_t + n_t \le (su_t * X_{SU}) \ , \forall \, t \quad \text{(S.4)}$$

$$0 \le h_t + bh_t \le X_H \ , \forall \, t \quad \text{(S.5)}$$

$$0 \le n_t + bn_t \le X_N \ , \forall \, t \quad \text{(S.6)}$$

$$bh_t \ge 0 \, , \forall \, t \quad \text{(S.7)}$$

$$bh_t \ge 0 \, , \forall \, t \quad \text{(S.8)}$$

$$0 \le e_t - D_{HB} \le X_{BP} \ , \forall \, t \quad \text{(S.9)}$$

$$0 \le hs_t \le X_{HS} \, , \forall \, t \quad \text{(S.10)}$$

$$0 \le bs_t \le X_{BS} \, , \forall \, t \quad \text{(S.11)}$$

$$0 \le ns_t \le X_{NS} \, , \forall \, t \quad \text{(S.12)}$$

$$bs_t = bs_{t-1} + e_t - D_{HB} - \ bn_t - bh_t, \forall \, t > 1 \quad \text{(S.13)}$$

$$hs_t = hs_{t-1} + h_t * Y + bh_t * Y - D_{H2} \, , \forall \, t > 1 \quad \text{(S.14)}$$

$$ns_t = ns_{t-1} + n_t * Z + bn_t * Z - D_{N2} \, , \forall \, t > 1 \quad \text{(S.15)}$$

$$bs_t = bs_0 + e_t - D_{HB} \ - \ bn_t - bh_t \, , t = 1 \quad \text{(S.16)}$$

$$hs_t = hs_0 + h_t * Y + bh_t * Y - D_{H2} \, , t = 1 \quad \text{(S.17)}$$

$$ns_t = ns_0 + n_t * Z + bn_t * Z - D_{N2} \, , t = 1 \quad \text{(S.18)}$$

$$0 \le bs_0 = bs_t \le X_{BS} \, , t = 8760 \quad \text{(S.19)}$$

$$0 \le hs_0 = hs_t \le X_{HS} \, , t = 8760 \quad \text{(S.20)}$$

$$0 \le ns_0 = ns_t \le X_{NS} \, , t = 8760 \quad \text{(S.21)}$$

In this case, the energy supply can now be allocated (Equation S.3) to hydrogen production (h_t), nitrogen production (n_t) or electricity (e_t), with the battery providing optional power for the hydrogen production (bh_t; Equations S.5) or nitrogen production (bn_t; Equation S.5). Additionally, the nitrogen generation capacity (X_N) and storage capacity (X_{NS}) and inventory (ns_t) are optimized variables. The energy demand for the ASU is separated from the HB process (D_{HB}) such that the power capacity of the battery is only the difference between electricity supply and HB demand (Equation S.9), assuming that the rate of recharging will be greater than the rate of discharging because the battery utilizes peaks in energy for recharging while providing power to a constant process. The nitrogen demand is capture in a separate parameter (D_{N2}), and the conversion of electricity into nitrogen is captured by the conversion factor *Z*.

In the case of a flexible HB process with ramping to align the production level with the fluctuations in renewable energy, the constant demand parameters D_{HB+ASU} & D_{H2} in Equations 5, 8 & 10 and Equations 9 & 11, respectively, become optimized variables with a time dependence ($d_{HB+ASU,t}$ & $d_{H2,t}$), but which operates on a different timescale τ. This is incorporated in the constraints as

$$0 \le e_t - d_{HB+ASU,\tau} \le X_{BP} \ , \forall \, t \quad \text{(S.22)}$$

$$bs_t = bs_{t-1} + e_t - d_{HB+ASU,\tau} \ , \forall \, t \quad \text{(S.23)}$$

$$hs_t = hs_{t-1} + h_t * Y - d_{H2,\tau}, \forall t \tag{S.24}$$

$$bs_t = bs_0 + e_t - d_{HB+ASU,\tau}, \forall t \tag{S.25}$$

$$hs_t = hs_0 + h_t * Y - d_{H2,\tau}, \forall t \tag{S.26}$$

$$\tau = \left\lceil \frac{t}{4} \right\rceil, \forall t$$

Additional constraints for the ramping of the HB process are described as

$$f_{min} * X_{HB+ASU} \leq d_{HB+ASU,\tau} \leq X_{HB+ASU} \tag{S.27}$$

$$-R * X_{HB+ASU} * 4 \leq d_{HB+ASU,\tau} - d_{HB+ASU,\tau-1} \leq R * X_{HB+ASU} * 4 \tag{S.28}$$

$$\frac{1}{2190} \sum_{\tau=1}^{2190} d_{HB+ASU,\tau} = D_{HB+ASU} \tag{S.29}$$

$$\frac{d_{HB+ASU,\tau}}{d_{H2,\tau}} = Z \tag{S.30}$$

where f_{min} is the minimum fractional capacity of the process, *R* is the maximum ramp rate of the process in percent of total capacity per hour and *Z* is the ratio of demand for electricity to demand for hydrogen, which is approximated to be constant as the production level of the HB process is adjusted.

iv. Cost and value allocation within an energy profile.

To calculate how the cost and value of energy differs among either a combination of profiles (e.g. solar and wind) or months of a year, the energy utilized in each stratum of an energy profile must be given hourly allocation over a year. Any given energy stratum is defined by the optimized hydrogen storage, electrolyser, battery storage and battery power capacity, and a profile of the energy utilized can be generated which is within the boundaries of the optimized process configuration. However, there are many different potential energy utilization profiles which fit the bounds of the optimization. Therefore, a method has been developed to distribute energy utilization (and curtailment) as evenly across a year as the bounds of the optimization will allow, and thus it assigns cost and value to energy as evenly as possible. This method is described in Algorithm 1.

Algorithm 1

Given:

SU_t^0: A vector of hourly power supply over a year

X_H, X_{HS}: Optimized electrolyser size, H2 storage size, and battery size and power for a given percent utilization

BC_t: A vector of hourly battery charging generated by linear optimization

Initialize:

CU_t: A vector of hourly energy curtailed over a year, initially zeros

D_t: A vector of hourly demand for *hydrogen* over a year (energy basis)

$SU_t = SU_t^0$: A vector of energy supply from which to remove energy through curtailment

A_t : A binary vector for each hour of the year describing if energy can be curtailed during that hour

$SU_t = SU_t - BC_t, \forall t$: Remove battery charging component from energy profile*

$CU_t = CU_t + (SU_t - X_H), for\ (SU_t - X_H) > 0$; Curtail energy due to exceeding X_H

$SU_t = X_H, for\ SU_t > X_H$

$SU_t^{CS} = \sum_1^t SU_t$, $SU_0^{CS} = 0$: Create vector of cumulative energy supply

$D_t^{CS} = \sum_1^t D_t$, $D_0^{CS} = 0$: Create vector of cumulative energy demand as hydrogen

$Sur = (SU_{end}^{CS} - SU_0^{CS}) - (D_{end}^{CS} - D_0^{CS})$: Calculate the current surplus over the year

While Sur > 0 :

$SUA_t = SU_t * A_t$, $\forall t$: Create vector of power available for curtailment

$SUA_t^{CS} = \sum_1^t SUA_t$, $SUA_0^{CS} = 0$: Create vector of cumulative available power

$$CU_{t1,t2}^{max} = \left[-1 * \left(SU_t^{CS} - SU_t^{CS^T}\right) + \left(D_t^{CS} - D_t^{CS^T}\right) + X_{HS}\right]$$
$$\oslash \left[-1 * \left(SUA_t^{CS} - SUA_t^{CS^T}\right)\right]$$

: Create a square matrix for the maximum percent curtailment (C) between any two hours in the energy profile, as determined by the optimal size of the hydrogen storage (X_{HS}). This equation is based on the general constraint:

$$Surplus_{t1\rightarrow t2} - Available\ Power_{t1\rightarrow t2} * Curtailment \geq -1 * X_{HS}$$

$CU_{t1,t2} = NaN$, $for\ CU_{t1,t2} < 0$: Remove negative percent curtailments

$CU^{limit} = \min_{t1,t2} CU_{t1,t2}^{max}$, $where\ CU_{t1,t2}^{max} = CU^{limit}\ at\ t1 = T1\ and\ t2 = T2$

: Find the limiting percent curtailment which can be applied

$If\ CU^{limit} > {Sur}/{(SUA_{end}^{CS} - SUA_0^{CS})}$:

$CU^{limit} = {Sur}/{(SUA_{end}^{CS} - SUA_0^{CS})}$

: If the maximum curtailment is greater than needed to close the energy balance, set the curtailment to that which closes the energy balance.

$CU_t = CU_t + SUA_t * CU^{limit}$, $\forall t$: Added curtailed energy to curtailment vector

$SU_t = SU_t - SUA_t * CU^{limit}$, $\forall t$: Remove energy from supply vector

$SU_t^{CS} = \sum_1^t SU_t$, $SU_0^{CS} = 0$: Update cumulative supply vector

$A_t = 0$, $for\ T1 < t \leq T2$

: Update the binary vector for power supply which can be curtailed

$Sur = (SU_{end}^{CS} - SU_0^{CS}) - (D_{end}^{CS} - D_0^{CS})$: Update the total surplus energy

$$E_m^{used} = {\sum_{t_{m,start}}^{t_{m,end}} (SU_t^0 - CU_t)} \Big/ {\sum_{t_{m,start}}^{t_{m,end}} (SU_t^0)}$$

: fraction of energy used in month m which runs from $t_{m,start}$ to $t_{m,end}$**

*It is assumed that the battery usage has no degrees of freedom because it is generally prioritized for recharging in the optimization algorithm, and it is a small amount of energy relative to hydrogen storage.

** In the case of differentiating the usage of energy between solar or wind energy, the fraction of the original energy supply at time t which is from solar or wind is added to this equation. The original energy supply and the amount of curtailment is then multiplied by this term such than the amount of solar or wind energy used in any hour is proportional to the fraction of solar or wind energy in that hour.

Algorithm 1 is repeat for all energy utilization levels for 1% to 100%. Algorithm 2 then calculates the LVOU and LAEC for each month using the LCOU and LAEC for each stratum within the energy profile, as defined from Equations 15 and 17, as well as the market value of ammonia (MV).

Algorithm 2

Given:

$E_{m,u}^{used}$: Vector of fraction of energy used in month m at utilization u

CU^{opt} : The optimal percent curtailment for a given location and year

$LCOU_s$: Cost of utilizing energy in stratum s

$LCOU_s^{INT}$: Cost of utilizing energy in stratum s based on the energy intermittency

C_{supply} : Energy supply cost defined as annual CapEx and OpEx per total energy

$$\Delta E_{m,s}^{used} = E_{m,u}^{used} - E_{m,u-1}^{m,used}\ , for\ s = u$$

: Calculate change in energy used in each month for each stratum

$$LVOU_m^{used} = \frac{\sum_{s=1}^{100-CU^{opt}} \left(\Delta E_{m,s}^{used} * (MV - LCOU_S)\right)}{\sum_{s=1}^{100-C\ \ opt} \Delta E_{m,s}^{used}}$$

: LVOU in month m of optimally used energy

$$LVOU_m^{total} = \frac{\sum_{s=1}^{100} \left(\Delta E_{m,s}^{used} * (MV - LCOU_S)\right)}{\sum_{s=1}^{100} \Delta E_{m,s}^{used}}$$

: LVOU in month m of all energy

$$LAEC_m^{used} = \frac{\sum_{s=1}^{100-CU^{opt}} \left(\Delta E_{m,s}^{used} * \left(C_{supply} * \left({}^{100}/_{100-CU^{opt}}\right) - LCOU_S^{INT}\right)\right)}{\sum_{s=1}^{100} \Delta E_{m,s}^{used}}$$

: LAEC in month m of optimally used energy

References

1 Vedde, J. *Photovoltaics*, Technology Data - Energy Plants for Electricity and District heating generation. 255-277 (Danish Energy Agency, 2015).

2 Sørensen, M. V., Petersen, H. S. & Nielsen, P. *Wind Turbines onshore*, Technology Data - Energy Plants for Electricity and District heating generation. 205-228 (Danish Energy Agency, 2016).

3 Nielsen, P. *Wind Turbines, Offshore*, Technology Data - Energy Plants for Electricity and District heating generation. 229-254 (Danish Energy Agency, 2016).

4 Martinez, A. & Iglesias, G. Mapping of the levelised cost of energy for floating offshore wind in the European Atlantic. *Renewable & Sustainable Energy Reviews* **154** (2022). https://doi.org/10.1016/j.rser.2021.111889

5 Sykes, V., Collu, M. & Coraddu, A. A Review and Analysis of the Uncertainty Within Cost Models for Floating Offshore Wind Farms. *Renewable & Sustainable Energy Reviews* **186** (2023). https://doi.org/10.1016/j.rser.2023.113634

6 Mayyas, A. T., Ruth, M. F., Pivovar, B. S., Bender, G. & Wipke, K. B. Manufacturing cost analysis for proton exchange membrane water electrolyzers. (National Renewable Energy Lab.(NREL), Golden, CO (United States), 2019).

7 Proost, J. State-of-the art CAPEX data for water electrolysers, and their impact on renewable hydrogen price settings. *International Journal of Hydrogen Energy* **44**, 4406-4413 (2019). https://doi.org/10.1016/j.ijhydene.2018.07.164

8 The Future of Hydrogen, Seizing Today's Opportunities, Report Prepared by the IEA for the G20, Japan. (Paris, France).

9 Cesaro, Z., Ives, M., Nayak-Luke, R., Mason, M. & Bañares-Alcántara, R. Ammonia to power: Forecasting the levelized cost of electricity from green ammonia in large-scale power plants. *Applied Energy* **282**, 116009 (2021). https://doi.org/10.1016/j.apenergy.2020.116009

10 Ikaheimo, J., Kiviluoma, J., Weiss, R. & Holttinen, H. Power-to-ammonia in future North European 100 % renewable power and heat system. *International Journal of Hydrogen Energy* **43**, 17295-17308 (2018). https://doi.org/10.1016/j.ijhydene.2018.06.121
11 Morgan, E. R. *Techno-Economic Feasibility Study of Ammonia Plants Powered by Offshore Wind*, University of Massachusetts Amherst, (2013).
12 Armijo, J. & Philibert, C. Flexible production of green hydrogen and ammonia from variable solar and wind energy: Case study of Chile and Argentina. *International Journal of Hydrogen Energy* **45**, 1541-1558 (2020). https://doi.org/10.1016/j.ijhydene.2019.11.028
13 Palys, M. J. & Daoutidis, P. Using hydrogen and ammonia for renewable energy storage: A geographically comprehensive techno-economic study. *Computers & Chemical Engineering* **136** (2020). https://doi.org/10.1016/j.compchemeng.2020.106785
14 Smith, C. & Torrente-Murciano, L. The importance of dynamic operation and renewable energy source on the economic feasibility of green ammonia. *Joule* **8** (2024). https://doi.org/10.1016/j.joule.2023.12.002
15 Mosbæk, R. R., Rao, M., Chochlidakis, C. & Rothuizen, R. *Hydrogen Storage*, Technology Data: Energy storage. 72-120 (Danish Energy Agency, 2020).
16 Mosbæk, R. R. & Jensen, S. H. *Lithium-ion batteries for grid-scale storage*, Technology Data: Energy storage. 162-188 (Danish Energy Agency, 2019).
17 Osman, O., Sgouridis, S. & Sleptchenko, A. Scaling the production of renewable ammonia: A techno-economic optimization applied in regions with high insolation. *Journal of Cleaner Production* **271** (2020). https://doi.org/10.1016/j.jclepro.2020.121627
18 Sánchez, A. & Martín, M. Optimal renewable production of ammonia from water and air. *Journal of Cleaner Production* **178**, 325-342 (2018). https://doi.org/10.1016/j.jclepro.2017.12.279
19 Ishimoto, Y. *et al.* Large-scale production and transport of hydrogen from Norway to Europe and Japan: Value chain analysis and comparison of liquid hydrogen and ammonia as energy carriers. *International Journal of Hydrogen Energy* **45**, 32865-32883 (2020). https://doi.org/10.1016/j.ijhydene.2020.09.017
20 Power to Ammonia: Feasibility Study for the Value Chains and Business Cases to Produce CO2-free Ammonia Suitable for Various Market Applications. (Amersfoort, The Netherlands, 2017).
21 Jensen, A. K. *Green Ammonia*, Technology Data: Renewable Fuels. 296-321 (Danish Energy Agency, 2021).
22 Guerra, C. F. *et al.* Technical-economic analysis for a green ammonia production plant in Chile and its subsequent transport to Japan. *Renewable Energy* **157**, 404-414 (2020). https://doi.org/10.1016/j.renene.2020.05.041
23 Egerer, J., Grimm, V., Niazmand, K. & Runge, P. The economics of global green ammonia trade - "Shipping Australian wind and sunshine to Germany". *Applied Energy* **334** (2023). https://doi.org/10.1016/j.apenergy.2023.120662
24 Bertuccioli, L. *et al.* Fuel cells and hydrogen Joint undertaking: Development of Water Electrolysis in the European Union. (2014).
25 Rouwenhorst, K. H. R., Ham, A. G. J. V. D., Mul, G. & Kersten, S. R. A. Islanded ammonia power systems : Technology review & conceptual process design. *Renewable & Sustainable Energy Reviews* **114** (2019). https://doi.org/10.1016/j.rser.2019.109339
26 Jin, R. C. *et al.* Controlling anisotropic nanoparticle growth through plasmon excitation. *Nature* **425**, 487-490 (2003). https://doi.org/10.1038/nature02020
27 *New European Wind Atlas*, (NEWA Consortium, 2022), <www.neweuropeanwindatlas.eu>.
28 Peterson, E. W. & Hennessey Jr, J. P. On the use of power laws for estimates of wind power potential. *Journal of Applied Meteorology Climatology* **17**, 390-394 (1978). https://doi.org/10.1175/1520-0450(1978)017<0390:OTUOPL>2.0.CO;2

29 Al-Quraan, A., Al-Masri, H., Al-Mahmodi, M. & Radaideh, A. Power curve modelling of wind turbines- A comparison study. *Iet Renewable Power Generation* **16**, 362-374 (2022). https://doi.org/10.1049/rpg2.12329

30 *2 MW Platform* (Vestas Wind Systems A/S, Denmark, 2023).

31 *GEBCO 2023 Grid*, (GEBCO Compilation Group, 2023). https://doi.org/10.5285/f98b053b-0cbc-6c23-e053-6c86abc0af7b
